# Impact of ambient air pollution on lung function in preterm-born school-aged children

**DOI:** 10.1136/thorax-2023-220233

**Published:** 2024-02-15

**Authors:** William John Watkins, Christopher William Course, Michael Cousins, Kylie Hart, Sarah J Kotecha, Sailesh Kotecha

**Affiliations:** 1 Department of Child Health, School of Medicine, Cardiff University, Cardiff, UK; 2 Department of Paediatrics, Cardiff & Vale University Health Board, Cardiff, UK

**Keywords:** Respiratory Measurement, Paediatric Lung Disaese, Clinical Epidemiology

## Abstract

**Rationale:**

Increased outdoor air pollution worsens lung function in children. However, these associations are less well studied in preterm-born individuals.

**Objectives:**

We assessed associations between ambient air pollutants and spirometry measures in preterm-born children.

**Methods:**

The Respiratory Health Outcomes in Neonates study recruited preterm-born children aged 7–12 years who were born at ≤34 week's gestation. We associated four ambient air pollutants (particulate matter with aerodynamic diameter ≤2.5 µm (PM_2.5_), PM_10_, nitrogen dioxide (NO_2_) and sulfur dioxide) at time of birth and spirometry assessment and averaged exposure between these two time points with spirometry measures, using linear regression analyses. Gestational age was banded into 23–28, 29–31 and 32–34 week's. Regression models estimated spirometry values against pollutant levels at birth and at the time of spirometry.

**Measurements and main results:**

From 565 preterm-born children, 542 (96%) had satisfactory data. After adjustments for early and current life factors, significant detrimental associations were noted between PM_10_ at birth and per cent predicted forced vital capacity (%FVC) for the 23–28 and 29–31 week's gestation groups and between current PM_2.5_ and NO_2_ exposure and %FVC for the 23–28 week's gestation group. No associations with spirometry were noted for the averaged pollution exposure between birth and spirometry. Predictive models showed 5.9% and 7.4% differences in %FVC between the highest and lowest current pollution exposures for PM_2.5_ and NO_2,_ respectively, in the 23–28 week group.

**Conclusions:**

Birth and current exposures to road-traffic-associated pollutants detrimentally affected %FVC in preterm-born school-aged children, who already have compromised lung function.

**WHAT IS ALREADY KNOWN ON THIS TOPIC**

- Ambient air pollution from industry and road traffic sources has been linked to poorer respiratory health and reduced lung function. However, the impact on preterm born individuals, a significant proportion of who already have compromised lungs, has yet to be studied.

**WHAT THIS STUDY ADDS**

- We have studied the effect of ambient air pollution at the time of birth and in childhood on spirometry measures in one of the largest contemporary preterm-born cohorts, finding links between elevated PM_10_, PM_2.5_ and nitrogen dioxide exposure and reduced per cent predicted forced vital capacity but not on other spirometry measures.

**HOW THIS STUDY MIGHT AFFECT RESEARCH, PRACTICE OR POLICY**

- Despite pollution levels being within recommended limits, this study highlights the need for further efforts to reduce ambient air pollution, as it has a significant impact on the respiratory function of the most preterm born individuals in later life.

## Introduction

It is well established that both antenatal and postnatal exposures to increased levels of air pollution have significant associations with childhood morbidity and mortality: our previous UK-based study demonstrated that sulfur dioxide (SO_2_), nitrogen dioxide (NO_2_) and particulate matter with aerodynamic diameter ≤10 µm (PM_10_) were differentially associated with all-cause neonatal and postneonatal mortality.^1^ Increased postnatal PM_10_ exposure has also been linked to higher risk of death from respiratory causes in children and adolescents.^2^ Aside from mortality, increased air pollution has also been linked to poorer respiratory health and lung function in childhood, although the relationships are not always clear. Antenatal exposure to PM_10_, especially that generated by road traffic, has been linked to decreased forced expiratory volume in 1 s (FEV_1_) and forced vital capacity (FVC) in 8-year-old offspring in the UK-based Avon Longitudinal Study of Parents and Children (ALSPAC) cohort.^3^ Postnatal exposure to NO_2_, PM_10_ and PM_2.5_ (PM with an aerodynamic diameter ≤2.5 µm) has also been differentially linked to significantly reduced FEV_1_ and FVC in childhood in several studies,^4–7^ with improvements in air quality over time associated with longitudinal increases in lung function.^8^ However, other studies have not demonstrated significant links between current air pollution and childhood lung function.^9 10^ Increased air pollution has been shown to be associated with worse lung function in individuals with underlying respiratory diseases. In children with asthma, exposure to SO_2_ has been associated with reduced FEV_1_ and FVC; and PM_10_ with reduced FEV_1_/FVC ratio and peak expiratory flow.^11^ In the large multicohort Evaluation Study of Congestive Heart Failure and Pulmonary Artery Catheterisation Effectiveness (ESCAPE) Study, long-term exposure to higher levels of NO_2_ and PM_10_ was associated with significant decreases in FEV_1_ and FVC in adults^12^ with a trend towards development of adult-onset asthma.^13^

Maternal exposure to high levels of outdoor air pollution appears to be a risk factor for preterm birth, especially at extremely preterm gestations.^14^ We and others have consistently shown that preterm birth, both with and without a diagnosis of bronchopulmonary dysplasia in infancy (BPD, also called chronic lung disease of prematurity), is associated with decreased lung function in childhood and beyond.^15–17^ Second trimester PM_10_ exposure has been shown to impact lung function at 44 week's postconceptional age in preterm-born infants, especially in those born at 32–36 week's gestation.^18^ For preterm-born infants with established BPD, living closer to major roadways following discharge home, and therefore, higher exposure to traffic-related air pollution, has been associated with increased respiratory morbidity.^19^ However, the impact of perinatal and childhood exposure to air pollution on the lung function of preterm-born school-aged children has been less well studied. We hypothesised that children born preterm, who were born at an immature stage of lung development with subsequent altered postnatal lung development,^20^ were at risk of decreased lung function when exposed to common ambient outdoor pollutants. We examined the relationships between SO_2_, NO_2_, PM_2.5_ and PM_10_ (a) at the time of birth, (b) current exposure at assessment and (c) averaged pollution exposure between birth and spirometry assessment with spirometry measures in children aged 7–12 years who were born at ≤34 week's gestation.

## Methods

This study was conducted on the cohort of preterm-born children recruited to the Respiratory Health Outcomes in Neonates study (RHiNO; EudraCT: 2015-003712-20), which has been previously described.^16 21^ Briefly, children recruited as part of a previous questionnaire study^22^ were supplemented with additional preterm-born children, sourced from the National Health Service Wales Informatics Service, and sent a respiratory and neurodevelopmental questionnaire, if they were born ≤34 week's gestation and aged 7–12 years old. Children were recruited from South Wales between 2016 and 2019. Children with significant congenital malformations, cardiopulmonary or neuromuscular disease were excluded.

Responders were invited to take part in a home or hospital-based assessment by two trained research nurses. Neonatal history and current health status were ascertained from questionnaire responses and corroborated with medical records. BPD was defined as supplemental oxygen-dependency for 28 days of age or greater for those born <32 week's gestation and at 56 days of age for those ≥32 week's gestation.^23^ Intrauterine growth restriction (IUGR) was defined as a birth weight <10th centile adjusted for gestational age and sex (LMS Growth v2.77, Medical Research Council, UK^24^). To evaluate contribution of non-respiratory neonatal illness, a severity score of zero or one, respectively, was created if none or one or more of the following diagnoses were present in the neonatal period: necrotising enterocolitis, intraventricular haemorrhage, retinopathy of prematurity and/or patent ductus arteriosus. Spirometry (Microloop, CareFusion, UK) was performed to American Thoracic Society (ATS)/European Respiratory Society (ERS) guidelines^25^ and normalised using Global Lung Initiative references to give per cent predicted lung function measures.^26^ Spirometry was performed at the time of recruitment. Averaged annual estimated measurements of four common air pollutants, PM_2.5_ PM_10_, NO_2_ and SO_2_, were obtained from the UK Government’s Department for Environment, Food and Rural Affairs (DEFRA) as previously reported,^1^ for the respective calendar year in which the child was born and for when spirometry was performed. The UK Government collected this data as part of the European Union’s Air Quality Directive from a network of monitoring stations throughout the UK, and verified by modelling to create pollutant levels for 1 km-by-1 km areas for each calendar year.^27^ To assess socioeconomic status, we used the most contemporaneous geographical assessed Welsh Index of Multiple Deprivation (WIMD) scores for 2014.^28^ The scores are calculated from composite measures of eight domains of deprivation including wealth, school achievement and home ownership. WIMD scores are given for 1909 geographical areas of similar population size (termed lower layer super output areas (LSOA), containing an average of approximately 1500 residents or 650 households). The LSOAs were supplied with x-coordinates and y-coordinates, on the same scale as the DEFRA 1 km-by-1 km grid, for its centre of mass—based on population distribution within the LSOA. The pollutant level for each combination of LSOA and time point was allocated by determining the closest, that is, minimum Euclidean distance, of the DEFRA grid points to each LSOA’s centre of mass to give pollutant exposure and WIMD scores for each participant at year of birth and at the time of spirometry.

Demographic variables were compared between groups using analysis of variance or χ^2^ pairwise comparisons as appropriate, both with Benjamini-Hochberg post hoc correction. The differential associations of each pollutant at time of birth, age at spirometry and averaged pollutant exposure scored between birth and current age with per cent predicted lung function measures (including %FEV_1_, %FVC, FEV_1_/FVC ratio and %FEF_25%–75%_ (forced expiratory flow at 25%–75% of FVC)) were tested through univariable and multivariable linear regression models. Participants were grouped into three gestational age bands: 23–28, 29–31 and 32–34 week's of gestation for analysis. The coefficients to be estimated were the estimands of main scientific interest, so gestation and pollution were included as an interaction term, whereby the beta values generated allowed direct comparison between the impact of pollution on lung function at the three gestation bands. Significant variables identified by univariable linear regression were visualised graphically in a directed acyclic graph. In the multivariable linear regression model, the continuous pollution-level variables and the categorical preterm group variable were included as interaction terms and in such a way as to cause the prematurity group variable to be treated like a dummy variable, consequently producing different pollution level coefficients for each prematurity group without requiring a reference category. This model was designed to include univariable linear regression results with p<0.1 for any spirometry variable, or early life factor including sex, BPD, IUGR and maternal age at birth (banded into six 5-year groups) and assessed the effect of gestational age in bands as an interaction term with pollutant level.

To explore the relationship between how different levels of ambient air pollution exposure at birth and at the current time of assessment would affect lung function values, an additional regression model was created, based on the univariable and multivariable linear regression results, to give estimated spirometry values at minimum and maximum pollutant exposures within this cohort. Those variables with p<0.1 for the univariable relationship with any of the lung function measures were combined into a multivariable linear model, including neonatal history of BPD, combined illness, IUGR and maternal age. Gestational band and raw pollutant level were included in a single interaction term to provide a simply interpretable beta coefficient for the effect of the pollutant at each gestation band. These models were used to calculate the predicted influence of the pollutant, at birth and at time of spirometry, over the full range of its measured values at these times on the lung function measures of %FEV_1_, %FVC, FEV_1_/FVC ratio and %FEF_25%–75%_ for each of the three gestational age bands. This included predicted spirometry values at minimum, mean and maximum pollutant exposure. All analyses were performed in SPSS V.25 (IBM) and R V.1.1.419.

## Results

From 565 preterm-born children taking part in the RHiNO study, 544 (96.3%) had valid spirometry data available as previously described.^16^ Two additional children were excluded, one for incorrect birth weight and one for incomplete address data. Since pollution data at time of birth was not available for six individuals due to changes in geographical boundaries, they were excluded from the ‘pollution exposure at the time of birth’ analyses only. The demographics for the included 542 children are shown in table 1. As expected, there was a significant difference between gestation groups for birth weight and rates of BPD, combined neonatal illness and wheeze-ever, with the most preterm-born group being affected the most. All lung function measures were significantly reduced in the most preterm-born group compared with the least preterm-born. Measures of deprivation and pollutant levels were not significantly different between the groups at birth or at time of spirometry (online supplemental tables 1 and 2, respectively). Table 2 shows the range of air pollutant levels within the study compared with contemporary recommended limits.

10.1136/thorax-2023-220233.supp1 Supplementary data

**Table 1:** Participant demographics

Variable	Gestation group (weeks)			Total (n=542)
	23–28 (n=99)	29–31 (n=165)	32–34 (n=278)	
Sex (male), n (%)	49 (49.5)	93 (56.4)	137 (49.3)	279 (51.5)
Birth weight (kg), mean (SD)	0.94 (0.24)	1.52 (0.39)	2.07 (0.44)***	1.70 (0.58)
IUGR, n (%)	10 (10.1)	21 (12.7)	27 (9.4)	58 (10.7)
Maternal age (age in years banded), n (%)				
≤19	9 (9.1)	7 (4.2)	4 (1.4)	20 (3.9)
20–24	18 (18.2)	23 (13.9)	25 (9.0)	66 (12.2)
25–29	22 (22.2)	37 (22.4)	72 (25.9)	131 (24.2)
30–34	24 (24.2)	49 (29.7)	90 (32.4)	163 (30.1)
35–39	19 (19.2)	37 (22.4)	73 (26.3)	129 (23.8)
≥40	7 (7.1)	12 (7.3)	14 (5.0)	33 (6.1)
BPD, n (%)	80 (80.8)***$$$	27 (16.4)***ˆˆˆ	1 (0.4)$$$ˆˆˆ	108 (19.9)
Combined neonatal illness, n (%)	54 (54.6) ***$$$	34 (20.6) ***ˆˆˆ	6 (2.2) $$$ˆˆˆ	94 (17.3)
Antenatal smoking, n (%)	14 (14.6)	17 (10.4)	31 (11.4)	62 (11.7)
Postnatal smoking, n (%)	17 (17.3)	30 (18.3)ˆ	27 (9.8)ˆ	74 (13.8)
Wheeze ever, n (%)	62 (65.3)$$	93 (57.4)	124 (47.0)$$	279 (53.6)
Wheeze in last 12 months, (%)	24 (24.2)	51 (30.9)	76 (27.3)	151 (27.9)
WIMD 2014 rank, mean (SD)	1122 (587)	1047 (558)	1046 (572)	1060 (570)
WIMD 2019 rank, mean (SD)	1124 (579)	1038 (556)	1059 (573)	1065 (569)
Age at spirometry (years), mean (SD)	10.2 (1.5)	10.1 (1.3)	10.2 (1.34)	10.1 (1.35)
%FEV_1_, mean (SD)	86.1 (12.2)*$$$	89.9 (12.3)*ˆˆ	93.6 (12.3)$$$ˆˆ	91.1 (12.6)
%FVC, mean (SD)	91.5 (10.6)$$	93.4 (10.1)	95.8 (12.2)$$	94.3 (11.4)
FEV_1_/FVC, mean (SD)	0.83 (0.08)$$	0.84 (0.08)	0.86 (0.07)$$	0.85 (0.08)
%FEF_25%–75%_, mean (SD)	69.1 (20.2)$$$	74.9 (21.6)ˆˆ	80.8 (19.2)$$$ˆˆˆ	76.9 (20.6)
Birth PM_2.5_ (µg/m^3^), mean (SD)	9.87 (1.3)	9.6 (1.25)	9.74 (1.15)	9.72 (1.21)
Birth PM_10_ (µg/m^3^), mean (SD)	15.54 (2.11)	15.13 (1.93)	15.37 (1.94)	15.33 (1.97)
Birth NO_2_ (µg/m^3^), mean (SD)	15.95 (5.44)	15.1 (6.02)	15.67 (5.8)	15.55 (5.8)
Birth SO_2_ (µg/m^3^), mean (SD)	3.08 (2.02)	2.8 (1.66)	3.05 (1.54)	2.98 (1.68)
Current PM_2.5_ (µg/m^3^), mean (SD)	8.03 (0.96)	7.89 (1.04)	8.04 (0.95)	7.99 (0.98)
Current PM_10_ (µg/m^3^), mean (SD)	12.49 (1.28)	12.39 (1.42)	12.57 (1.35)	12.5 (1.36)
Current NO_2_ (µg/m^3^), mean (SD)	13.27 (4.59)	12.85 (5.34)	13.67 (5.04)	13.35 (5.06)
Current SO_2_ (µg/m^3^), mean (SD)	1.98 (0.57)	2.06 (0.77)	2.1 (0.8)	(0.75)

Combined neonatal illness: at least one of Necrotising enterocolitis (NEC), Intraventricular haemorrhage (IVH), Retinopathy of prematurity (ROP), Patent ductus arteriosus (PDA).

23–28 vs 29–31: *p<0.05, **p<0.01, ***p<0.001; 23–28 vs 32–34: $p<0.05, $$p<0.01, $$$p<0.001; 29–31 vs 32–34: ˆp<0.05, ˆˆp<0.01, ˆˆˆp<0.001 by ANOVA/χ2 with post hoc Benjamini-Hochberg for multiple pairwise comparisons.

ANOVA, analysis of variance; BPD, bronchopulmonary dysplasia.; %FEF_25%–75%_, forced expiratory flow at 25%–75% of forced vital capacity; %FEV_1_, per cent predicted forced expiratory volume in 1 s; %FVC, per cent predicted forced vital capacity; IUGR, intrauterine growth restriction; NO2, nitrogen dioxide; PM_10_, particulate matter with aerodynamic diameter ≤10 µm; PM_2.5_, particulate matter with aerodynamic diameter ≤2.5 µm;; SO2, sulfur dioxide; WIMD, Welsh Index of Multiple Deprivation.

**Table 2:** Ambient air pollution levels in study and recommended annual exposure limits

	PM_2.5_	PM_10_	SO_2_	NO_2_
Birth, mean (range), (µg/m^3^)	9.72 (6.90–13.31)	15.33 (10.10–20.56)	2.98 (0.81–15.89)	15.55 (4.48–34.93)
Current, mean (range), (µg/m^3^)	8.00 (5.58–10.58)	12.50 (8.83–16.31)	2.07 (0.55–8.82)	13.41 (3.80–29.24)
European Union limit value 2008, (µg/m^3^)	25	40	20*	40
WHO guideline value 2005, (µg/m^3^)	10	20	20†	40

*European Union directive gives an annual exposure limit for protection of vegetation, no annual exposure level recommended for protection of human health.

†WHO average 24-hour limit.

NO_2_, nitrogen dioxide; PM_10_, particulate matter with aerodynamic diameter ≤10 µm; PM_2.5_, particulate matter with an aerodynamic diameter ≤2.5 µm; SO_2_, sulfur dioxide.

Results of univariable regression analyses are shown in table 3. Significant negative associations were noted between spirometry variables and demographic variables including gestational age band (%FEV_1_, %FVC, FEV_1_/FVC ratio and %FEF_25%–75%_), BPD (%FEV_1_, %FVC, FEV_1_/FVC ratio and %FEF_25%–75%_), combined neonatal illness (%FEV_1_, %FVC, FEV_1_/FVC ratio and %FEF_25%–75%_), younger maternal age at birth (%FEV_1_, %FVC, FEV_1_/FVC ratio and %FEF_25%–75%_) and history of IUGR (%FEV_1_, %FEF_25%–75%_). These are represented graphically in a directed acyclic graph in online supplemental figure 1. No associations were noted between lung function measures and antenatal/postnatal smoking or deprivation. A significant association was observed between PM_10_ level at birth and %FVC (β=−0.52, p=0.036).

**Table 3:** Univariable linear regression analyses of spirometry measures

Variable	%FEV_1_		%FVC		FEV_1_/FVC ratio		%FEF_25%–75%_	
	β (95% CI)	P value	β (95% CI)	P value	β (95% CI)	P value	β (95% CI)	P value
Sex (ref=female)	1.129 (−0.996 to 3.253)	0.296	1.127 (−0.794 to 3.048)	0.248	0.024 (0.011 to 0.037)	<0.001	−1.241 (−4.723 to 2.241)	0.483
IUGR (ref=no)	−4.842 (−8.256 to 1.428)	0.005	−2.767 (−5.867 to 0.334)	0.079	−0.016 (−0.037 to 0.005)	0.136	−8.741 (−14.325 to 3.157)	0.002
Gestational age group (weeks)								
23–28	−7.461 (−10.285 to 4.637)	<0.001	−4.253 (−6.847 to 1.66)	0.001	−0.032 (−0.049 to 0.014)	<0.001	−11.791 (−16.424 to 7.158)	<0.001
29–31	−3.645 (−6.016 to 1.274)	0.002	−2.324 (−4.502 to 0.146)	0.036	−0.017 (−0.032 to 0.003)	0.019	−5.957 (−9.847 to 2.067)	0.003
32–34 (ref)	ref		ref		ref		ref	
Mother age band (years)								
≤19	−9.751 (−15.553 to 3.949)	0.001	−4.071 (−9.36 to 1.217)	0.128	−0.053 (−0.089 to 0.017)	0.003	−16.291 (−25.822 to 6.761)	0.001
20–24	−4.316 (−7.889 to 0.743)	0.017	−3.446 (−6.702 to 0.189)	0.037	−0.007 (−0.029 to 0.015)	0.548	−3.776 (−9.645 to 2.092)	0.204
25–29	−1.972 (−4.846 to 0.901)	0.175	−0.873 (−3.492 to 1.747)	0.51	−0.007 (−0.025 to 0.01)	0.403	−2.7 (−7.42 to 2.02)	0.259
30–34 (ref)	ref		ref		ref		ref	
35–39	−2.386 (−5.272 to 0.5)	0.102	−1.211 (−3.842 to 1.419)	0.363	−0.005 (−0.022 to 0.013)	0.602	−4.039 (−8.78 to 0.701)	0.092
≥40	−4.452 (−9.127 to 0.222)	0.060	−3.097 (−7.358 to 1.164)	0.151	−0.014 (−0.042 to 0.015)	0.342	−3.973 (−11.652 to 3.705)	0.307
BPD (ref=no)	−6.08 (−8.691 to 3.469)	<0.001	−2.873 (−5.267 to 0.479)	0.018	−0.031 (−0.047 to 0.015)	<0.001	−10.227 (−14.5 to 5.955)	<0.001
Combined neonatal illness (ref=no)	−6.963 (−9.715 to 4.21)	<0.001	−3.861 (−6.385 to 1.338)	0.003	−0.031 (−0.048 to 0.014)	<0.001	−11.166 (−15.694 to 6.638)	<0.001
Antenatal smoking (ref=no)	−0.364 (−3.718 to 2.991)	0.831	−0.583 (−3.61 to 2.443)	0.704	0 (−0.021 to 0.02)	0.983	−0.01 (−5.505 to 5.485)	0.997
Postnatal smoking (ref=no)	−0.886 (−3.986 to 2.213)	0.574	−0.139 (−2.943 to 2.665)	0.922	−0.008 (−0.027 to 0.011)	0.402	−2.714 (−7.788 to 2.36)	0.292
NO_2_ (birth)	0.012 (−0.172 to 0.197)	0.896	−0.063 (−0.23 to 0.104)	0.455	0.001 (0 to 0.002)	0.184	0.094 (−0.205 to 0.393)	0.535
SO_2_ (birth)	0.095 (−0.544 to 0.734)	0.771	0.101 (−0.477 to 0.679)	0.731	0 (−0.004 to 0.004)	0.973	−0.015 (−1.049 to 1.02)	0.978
PM_2.5_ (birth)	−0.177 (−1.06 to 0.707)	0.694	−0.446 (−1.244 to 0.352)	0.272	0.003 (−0.003 to 0.008)	0.345	0.137 (−1.293 to 1.567)	0.85
PM_10_ (birth)	−0.267 (−0.81 to 0.276)	0.333	−0.522 (−1.011 to 0.032)	0.036	0.001 (−0.002 to 0.004)	0.579	0.192 (−0.687 to 1.071)	0.667
NO_2_ (current)	−0.064 (−0.275 to 0.146)	0.548	−0.17 (−0.36 to 0.019)	0.078	0.001 (0 to 0.002)	0.14	0.088 (−0.256 to 0.433)	0.615
SO_2_ (current)	0.064 (−1.349 to 1.476)	0.93	−0.486 (−1.763 to 0.79)	0.455	0.006 (−0.003 to 0.014)	0.206	1.092 (−1.221 to 3.404)	0.354
PM_2.5_ (current)	−0.454 (−1.539 to 0.631)	0.412	−0.907 (−1.886 to 0.072)	0.069	0.004 (−0.003 to 0.01)	0.257	0.331 (−1.448 to 2.11)	0.715
PM_10_ (current)	−0.098 (−0.882 to 0.685)	0.805	−0.508 (−1.216 to 0.199)	0.159	0.004 (−0.001 to 0.009)	0.108	0.516 (−0.767 to 1.798)	0.43
WIMD (birth)								
1 (most deprived)	1.157 (−2.12 to 4.434)	0.486	0.901 (−2.062 to 3.865)	0.548	0.003 (−0.017 to 0.023)	0.771	2.186 (−3.106 to 7.478)	0.415
2	0.865 (−2.256 to 3.985)	0.584	1.292 (−1.53 to 4.114)	0.366	−0.004 (−0.023 to 0.015)	0.689	0.959 (−4.08 to 5.998)	0.707
3	0.59 (−2.608 to 3.788)	0.716	1.768 (−1.124 to 4.659)	0.228	−0.012 (−0.032 to 0.007)	0.213	−0.796 (−5.96 to 4.368)	0.761
4	−0.718 (−4.139 to 2.702)	0.679	0.828 (−2.266 to 3.921)	0.597	−0.017 (−0.038 to 0.003)	0.098	−3.268 (−8.792 to 2.255)	0.243
5 (least deprived) (ref)	Ref		Ref		Ref		Ref	
WIMD (current)								
1 (most deprived)	0.483 (−2.817 to 3.783)	0.774	−0.791 (−3.774 to 2.192)	0.603	0.014 (−0.006 to 0.034)	0.172	3.306 (−2.088 to 8.699)	0.229
2	1.605 (−1.615 to 4.824)	0.328	2.153 (−0.757 to 5.063)	0.147	−0.004 (−0.023 to 0.016)	0.698	1.207 (−4.055 to 6.469)	0.653
3	−0.727 (−3.748 to 2.295)	0.637	−0.009 (−2.74 to 2.722)	0.995	−0.008 (−0.026 to 0.011)	0.423	−0.651 (−5.589 to 4.287)	0.796
4	1.903 (−1.41 to 5.215)	0.260	0.281 (−2.713 to 3.275)	0.854	0.011 (−0.01 to 0.031)	0.302	5.175 (−0.238 to 10.589)	0.061
5 (least deprived) (ref)	Ref		Ref		Ref		Ref	

Combined neonatal illness: History of any of the following neonatal diagnoses; necrotising enterocolitis, intraventricular haemorrhage, retinopathy of prematurity, patent ductus arteriosus.

BPD, bronchopulmonary dysplasia; %FEF_25%–75%_, forced expiratory flow at 25%–75% of forced vital capacity; %FEV_1_, per cent predicted forced expiratory volume in 1 ss; %FVC, per cent predicted forced vital capacity; IUGR, intrauterine growth restriction; NO_2_, nitrogen dioxide; PM_10_, particulate matter with aerodynamic diameter ≤10 µm; PM_2.5_, particulate matter with aerodynamic diameter ≤2.5 µm; SO_2_, sulfur dioxide; WIMD, Welsh Index of Multiple Deprivation.

**Figure 1:**
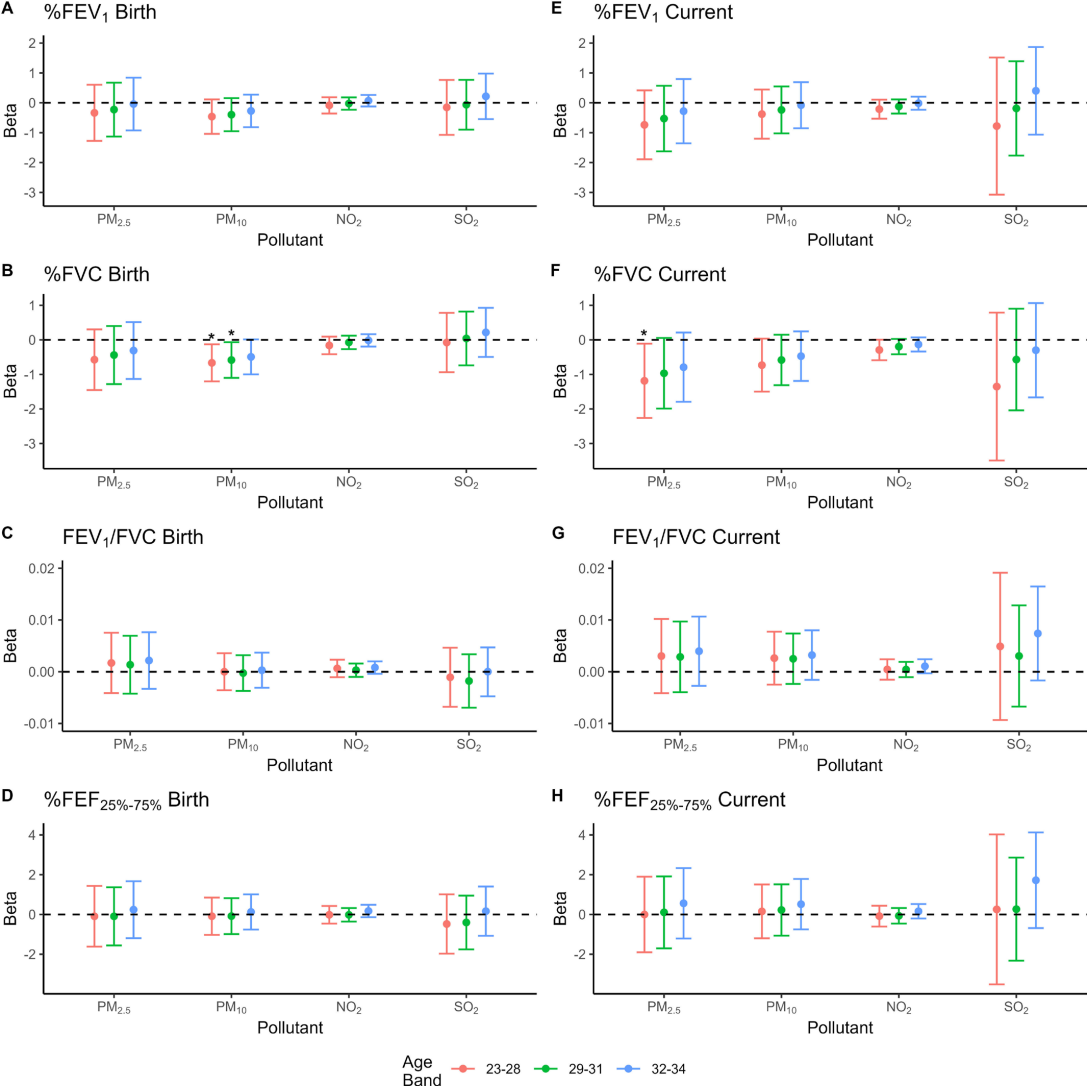
Plot of beta-values and 95% CIs from multivariable linear regression models of lung function parameters and pollutant exposures by each gestational age band (adjusted for sex, IUGR, BPD, combined neonatal illness and maternal age). Starred values represent betas with a p<0.05. BPD, bronchopulmonary dysplasia; %FEV_1_, per cent predicted forced expiratory volume in 1 s; %FVC, per cent predicted forced vital capacity; %FEF_25%–75%_, forced expiratory flow at 25%–75% of forced vital capacity; IUGR, intrauterine growth restriction; NO_2_, nitrogen dioxide; PM_2.5_, particulate matter with aerodynamic diameter ≤ 2.5µm; PM_10_, particulate matter with aerodynamic diameter ≤10 µm; SO_2_, sulfur dioxide.

Figure 1 and online supplemental table 3 show the results of the multivariable regressions. Significant negative associations were noted between %FVC for those born at 23–28 (β=−0.66, p=0.016) and at 29–31 week's gestation (−0.58, p=0.027) and PM_10_ exposure at birth. The %FVC results for those born at 32–34 week's gestation had β of −0.49 and p value of 0.058 for PM_10_ exposure at birth. Current exposure to PM_2.5_ and NO_2_ had a negative association for %FVC in the 23–28 week's gestation group (−1.19, p=0.031; −0.29, p=0.05, respectively). The associations for the 29–31 week's gestation group and PM_2.5_ and NO_2_ were β of −0.92 and p value of 0.070 and β of −0.19 and p value of p=0.083, respectively). No significant associations were noted between the pollutants and %FEV_1_ or %FEF_25%–75%_. The level of ambient air pollution decreased between the two time points measured. We did not observe any significant associations between the calculated mean pollutant exposure between these two time points (birth and current exposure levels) for PM_2.5_, PM_10_, NO_2_ or SO_2_ and any spirometry measure (online supplemental table 4).

**Figure 2:**
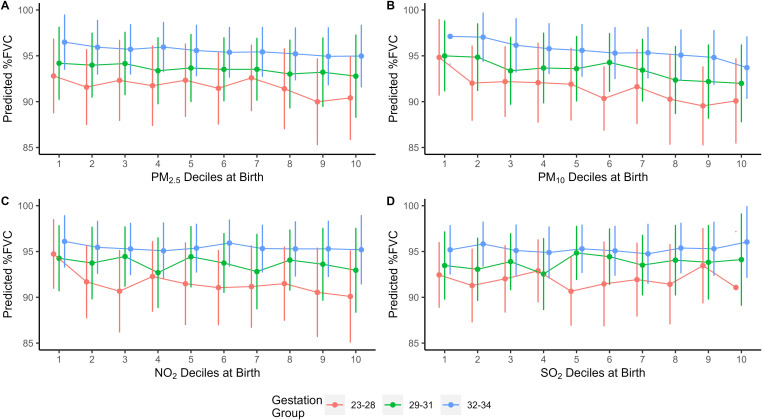
(A–D) Per cent predicted FVC values for each gestational age group from adjusted regression model by each pollutant decile at birth. Points represent mean value. Vertical bars represent 95% CI. FVC, forced vital capacity; NO_2_, nitrogen dioxide; PM_2.5_, particulate matter with aerodynamic diameter ≤ 2.5µm; PM_10_, particulate matter with aerodynamic diameter ≤10 µm; SO_2_, sulfur dioxide.

**Figure 3:**
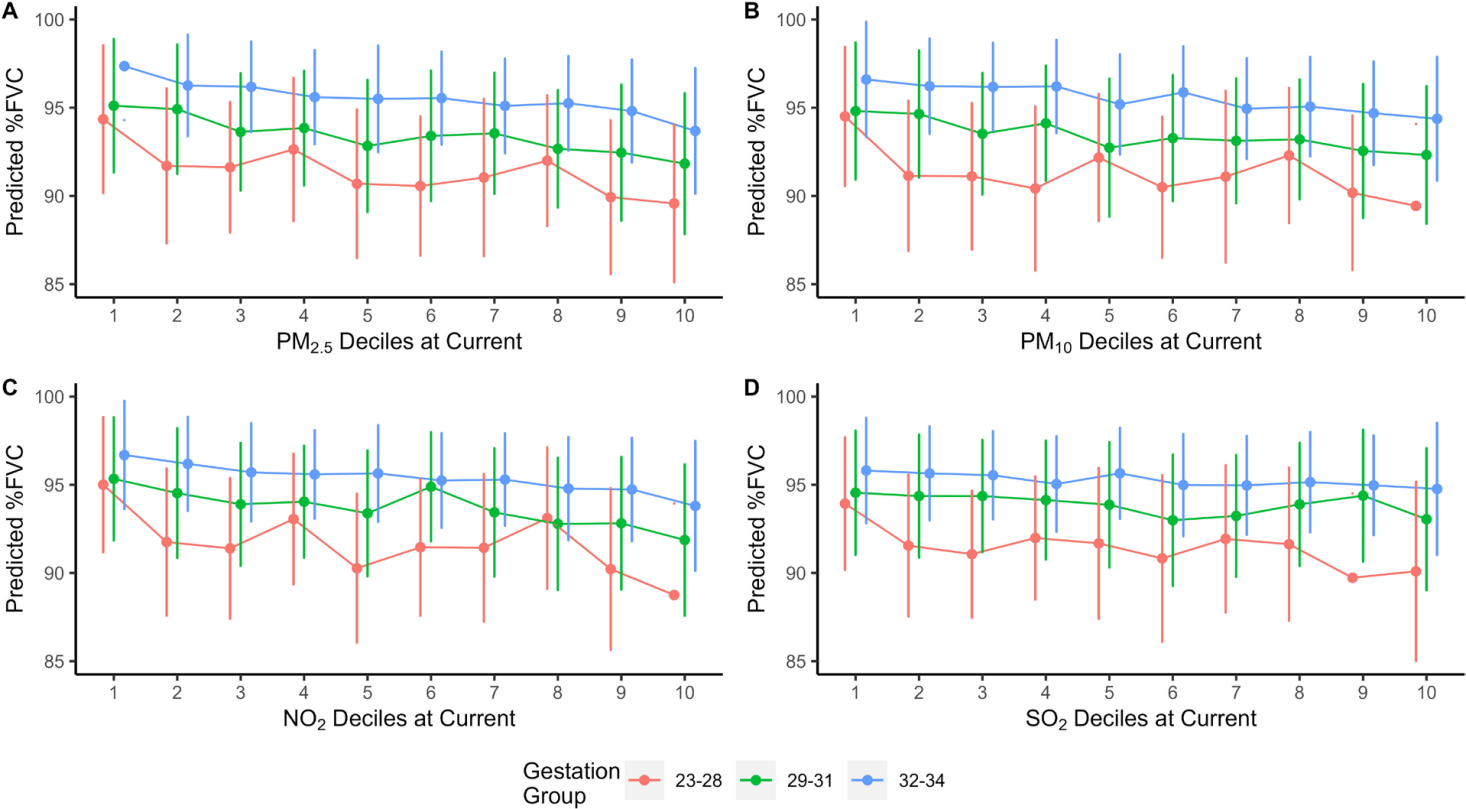
(A–D) Per cent predicted FVC values for each gestational age group from adjusted regression model by each pollutant decile at current exposure. Points represent mean value. Vertical bars represent 95% CI. FVC, forced vital capacity; NO_2_, nitrogen dioxide; PM_2.5_, particulate matter with aerodynamic diameter ≤ 2.5µm; PM_10_, particulate matter with aerodynamic diameter ≤10 µm; SO_2_, sulfur dioxide.

As described in the methods, additional regression models were created based on the univariable and multivariable linear regression results for estimating spirometry values over the total range of pollutant exposures within this cohort. The results from these models for birth and current pollution exposure and %FVC are shown in table 4 and figures 2 and 3; and in online supplemental file 1 for %FEV_1_, FEV_1_/FVC ratio and %FEF_25%–75%_ (online supplemental tables 5–7 and figures 2–4, respectively). Online supplemental table 8 gives the decile measurement for each pollutant. PM_10_ exposure at birth was associated with significant decrease in %FVC for those born 23–28 week's and 29–31 week's gestation, with 6.95% (p=0.02) and 6.1% (p=0.03) reduction in %FVC, respectively, between lowest and highest PM_10_ exposures. No other significant associations were noted for %FVC and pollutant levels at time of birth. No significant differences were seen for %FEV_1_, FEV_1_/FVC and %FEF_25%–75%_.

**Table 4:** Regression model for FVC values by gestational age band over minimum to maximum exposure to PM_2.5_, PM_10_, SO_2_ and NO_2_ at birth and current time points

Variable		For minimum	For mean	For maximum	Min–Max difference	Beta (95% CI)	P value
		Pollutant exposure	Pollutant exposure	Pollutant exposure	(% of mean)		
Birth PM_2.5_ (µg/m^3^)		6.9	9.72	13.31	6.41 (65.94)		
%FVC by gestation (weeks)	23–28	93.11	91.49	89.44	3.68 (4.02)	−0.57 (−1.45 to 0.3)	0.2
	29–31	94.67	93.42	91.85	2.82 (3.02)	−0.44 (−1.28 to 0.4)	0.31
	32–34	96.62	95.75	94.64	1.98 (2.07)	−0.31 (−1.13 to 0.52)	0.46
Birth PM_10_ (µg/m^3^)		10.1	15.33	20.56	10.46 (68.26)		
%FVC by gestation (weeks)	23–28	94.97	91.49	88.02	6.95 (7.6)	−0.66 (−1.2 to 0.13)	0.02
	29–31	96.47	93.42	90.37	6.1 (6.53)	−0.58 (−1.1 to 0.07)	0.03
	32–34	98.32	95.75	93.17	5.15 (5.37)	−0.49 (−1 to 0.02)	0.06
Birth SO_2_ (µg/m^3^)		0.81	2.98	15.89	15.08 (505.82)		
%FVC by gestation (weeks)	23–28	91.66	91.49	90.51	1.15 (1.26)	−0.08 (−0.94 to 0.78)	0.86
	29–31	93.33	93.42	93.96	−0.62 (−0.67)	0.04 (−0.74 to 0.82)	0.92
	32–34	95.28	95.75	98.55	−3.27 (−3.42)	0.22 (−0.5 to 0.93)	0.55
Birth NO_2_ (µg/m^3^)		4.48	15.55	34.93	30.45 (195.81)		
%FVC by gestation (weeks)	23–28	93.27	91.49	88.38	4.89 (5.35)	−0.16 (−0.42 to 0.09)	0.22
	29–31	94.23	93.42	92.01	2.23 (2.38)	−0.07 (−0.27 to 0.12)	0.46
	32–34	95.9	95.75	95.48	0.42 (0.44)	−0.01 (−0.19 to 0.17)	0.88
Current PM_2.5_ (µg/m^3^)		5.58	7.99	10.58	5.01 (62.66)		
%FVC by gestation (weeks)	23–28	94.36	91.49	88.42	5.94 (6.49)	−1.19 (−2.26 to 0.11)	0.03
	29–31	95.76	93.42	90.92	4.84 (5.18)	−0.97 (−1.99 to 0.06)	0.06
	32–34	97.66	95.75	93.7	3.96 (4.13)	−0.79 (−1.79 to 0.21)	0.12
Current PM_10_ (µg/m^3^)		8.83	12.5	16.31	7.48 (59.84)		
%FVC by gestation (weeks)	23–28	94.18	91.49	88.71	5.47 (5.98)	−0.73 (−1.5 to 0.04)	0.06
	29–31	95.56	93.42	91.21	4.35 (4.66)	−0.58 (−1.31 to 0.15)	0.12
	32–34	97.48	95.75	93.96	3.52 (3.68)	−0.47 (−1.19 to 0.25)	0.2
Current SO_2_ (µg/m^3^)		0.55	2.07	8.82	8.27 (400.36)		
%FVC by gestation (weeks)	23–28	93.55	91.49	82.37	11.18 (12.22)	−1.35 (−3.49 to 0.79)	0.22
	29–31	94.29	93.42	89.58	4.7 (5.03)	−0.57 (−2.04 to 0.9)	0.45
	32–34	96.2	95.75	93.73	2.47 (2.58)	−0.3 (−1.67 to 1.07)	0.67
Current NO_2_ (µg/m^3^)		3.8	13.35	29.24	25.44 (190.6)		
%FVC by gestation (weeks)	23–28	94.29	91.49	86.85	7.44 (8.13)	−0.29 (−0.59 to 0)	0.05
	29–31	95.28	93.42	90.33	4.95 (5.3)	−0.19 (−0.42 to 0.03)	0.08
	32–34	97	95.75	93.67	3.33 (3.48)	−0.13 (−0.34 to 0.07)	0.21

Regression model for %FVC values over range of current pollutant exposures measured. Beta and significance level for relationship between difference in lung function and pollutant levels for each gestational age band.

%FVC, per cent predicted forced vital capacity; NO_2_, nitrogen dioxide; PM_10_, particulate matter with aerodynamic diameter ≤10 µm; PM_2.5_, particulate matter with aerodynamic diameter ≤2.5 µm; SO_2_, sulfur dioxide.

There was a difference for %FVC of 5.94% (p=0.03) for current PM_2.5_ and 7.44% (p=0.05) for current NO_2_ exposure for preterm-born children born at 23–28 week's gestation when the lowest and highest exposures were compared. The predictive models did not demonstrate any significant differences between %FVC and SO_2_ at time of spirometry for any gestational group. No significant differences were noted for current exposure levels and predicted differences in %FEV_1_, FEV_1_/FVC and %FEF_25%–75%_ between the lowest and highest exposures.

## Discussion

In this study of preterm-born children, we have shown that current exposure to increasing levels of two major ambient air pollutants, PM_2.5_ and NO_2_, was associated with significantly decreased %FVC in a graded manner with decreasing gestational age. We have also reported that increased PM_10_ exposure at the time of birth was associated with reduced %FVC in the same gestationally graded manner as postnatal exposure. The averaged exposure between birth and spirometry assessment was not associated with any of the spirometry measures. However, we did not note any association between pollution levels at any time point with either %FEV_1_ or %FEF_25%–75%_. Furthermore, birth, current and averaged SO_2_ exposures were not associated with any spirometry measures. We generated adjusted regression models which showed significant relationships between %FVC and PM_10_ for exposures at birth; and between %FVC and PM_2.5_/NO_2_for current pollution exposures. These effects occurred in a gestationally graded manner with the most prematurely born children affected the most.

Increased ambient outdoor air pollution has been strongly associated with increased respiratory morbidity and mortality in adult populations.^29^ Our previous study suggested that both antenatal and postnatal exposure of the four pollutants studied here can affect both neonatal and postneonatal mortality possibly via antenatal maternal exposure and postnatal exposure.^1^ Preterm-born infants have previously been shown to be more susceptible to the detrimental effects of antenatal exposure than term-born infants, with increasing antenatal exposure to PM_10_ associated with worse lung function and higher fractional exhaled nitric oxide at 44 week's postmenstrual age.^18^ In addition, antenatal exposure to increased PM_10_ has been demonstrated to have a detrimental impact on placental growth and function,^30^ and on birth weight.^31 32^ Our current data have demonstrated that perinatal exposure to PM_10_ could have lasting effects resulting in decreased %FVC in children who were born at <32 week's gestation. These infants are likely to be at higher risk of airway epithelial injury which could potentially explain the detrimental effects we observed for pollutants on %FVC via the resultant injury on the lung parenchyma. This association is in keeping with findings from the ALSPAC cohort^3^ which noted an association between antenatal PM_10_ maternal exposure and reduced FVC in 8-year-old offspring who were, however, born at term. Unlike the ALSPAC study, we did not observe an association between pollutants and %FEV_1_. The reasons for this are unclear. It is known that larger airways are compromised after preterm birth.^17^ Whether these airways are already so compromised in early infancy, especially in the lowest gestation group, such that additional epithelial injury from pollutant exposure has little further effect is very speculative and needs further study.

Several previous studies, largely of term-born children, have also reported that outdoor air pollution exposure has a negative impact on lung function in paediatric and adult populations, but whether such exposure affects preterm-born children is less known. A Swedish study reported that high exposure to traffic-related air pollution (nitrogen oxides (NO_x_) and PM_10_) in the first year of life was associated with decreased FEV_1_ and FVC^33^ and, although NO_x_ and PM_10_ in the same cohort were associated with reduced FEV_1_ at 8 years of age, no association was noted between pollutant exposure and FVC.^34^ A Norwegian study examined NO_2_, PM_2.5_ and PM_10_ exposure in the first 2 years of life and in children up to 9–10 years noting a strong association between increasing air pollution exposure and reduced forced expiratory flows (peak expiratory flow, FEF_25%_ and FEF_50%_) but no associations were noted for FVC. Longer-term exposure to high air pollution had a stronger association with reduced expiratory flows.^35^ Another California-based study examined the effects of air pollution on changes of lung function over an 8-year period in children aged 10–18. They noted that exposure to higher levels of NO_2_ and acid vapour (a mixture of inorganic and organic acids, primarily produced by motor engines) resulted in slower growth of FVC over the study period, on average being 95 mL and 105 mL, respectively, lower than those exposed to low air pollution. Significant reduction in the growth of FEV_1_ was also associated with higher exposure to NO_2_, acid vapour and PM_2.5_, with a near-significant association with PM_10_.^36^ A recent Chinese study demonstrated that long-term exposure to PM_2.5_ had a negative impact on lung function in children aged 7–12 years, with a one unit increase in the average daily dose exposure to PM_2.5_ resulting in 14.8 mL decrease in FVC and a 10 mL reduction in FEV_1_.^37^ Why there are differences between cohorts for spirometry measures for the common pollutants is unclear especially as the pollutants’ exposures were similar between the cohorts. We did not see a significant relationship between FEV_1_ and pollution exposure in our cohort and the reason for this is unclear from our data. We can hypothesise that the degree of FEV_1_ impairment occurring solely as a consequence of preterm birth, which we have previously demonstrated,^17^ is so significant that the impact of ambient air pollution on FEV_1_ in this population is masked. Additional studies, especially using personal exposure devices, are required to determine if FVC is commonly affected in all preterm-born cohorts.

Road traffic is the primary source of PM_2.5_, PM_10_ and NO_2_ air pollution especially in urban areas, primarily from petrol-engine and diesel-engine exhaust fumes, and from vehicle tyre and brake wear. SO_2_ is more associated with industrial activity and power station emissions.^38^ We noted an improvement in air pollution levels between birth and time of spirometry, with the majority of pollution levels being below European Union legal limits and WHO guidelines.^38^ However, even within these recommended limits, we have noted air pollution continues to have a detrimental association with FVC. This highlights the paucity of evidence on what constitutes a ‘safe’ level of air pollution exposure. As many of the associations were seen between FVC and PM_2.5_, PM_10_ and NO_2_, this highlights the importance of concerted efforts to reduce road traffic and fossil-fuel burning vehicle engines in urban areas to prevent further detrimental effects on preterm-born children’s respiratory function. We did not see a significant association between deprivation and lung function in univariable modelling for our cohort, however, deprivation has been linked to higher air pollution exposure,^39^ respiratory morbidity and decline in lung function^40 41^ in other studies.

Infants delivered at extremely preterm gestations (23–28 week's gestation) are born at an immature stage of lung development, at late canalicular and early saccular phases.^42^ Subsequent ex utero aberrant lung development, partially due to postnatal interventions such as respiratory support and increased supplemental oxygen therapy, then results in structurally and functionally altered lungs for these infants in later life resulting in significant respiratory morbidity.^17 20^ Our data showed that children born at extremes of gestational immaturity were most susceptible to the negative effects of several ambient outdoor air pollutants and may further impact lung growth as the primary effect we have observed was on FVC. Although the changes in lung function between lowest and highest pollution levels may seem small in childhood, these trajectories are likely to enhance with time given that lung function tracks over time^43^ with consequent early development of chronic obstructive pulmonary disease as recently postulated.^44^ Therefore, the negative effects on lung function of higher levels of ambient outdoor air pollution on this already vulnerable population cannot be ignored. Higher FVC, a proxy measure of lung size, has previously been shown to be positively associated with increased life expectancy in adults,^45^ and therefore, this already vulnerable population may have increased early respiratory mortality if exposed to high levels of air pollution.

Strengths of this study include a large preterm-born cohort who represent a wide range of gestational ages at birth, with good representation of extremely preterm-born subjects, which permitted us to delineate the different associations of air pollution and lung function for individuals born at a range of different stages of lung development. We had comprehensive antenatal, neonatal and postnatal medical histories, which allowed us to model our results including important neonatal comorbidities such as BPD and IUGR. We used robustly modelled, publicly available pollution data, which allowed us to accurately ascertain individual participants exposures both at birth and at time of current assessment. Limitations include that we studied ambient outdoor air pollution but not indoor air pollution exposures, which may further adversely impact on lung function. Our measures of pollution exposures were based on small geographical area exposures and not on personal exposure thus may impact on our findings especially if localised exposures were either enhanced or reduced. Similarly, measures of deprivation, such as WIMD, are proxy measures of socioeconomic status based on geographical location. Even in small areas exposure may differ between close neighbourhoods, for instance, due to proximity to major roads or highways. Smoking status was self-reported by parents and may have been under-reported, explaining the lack of association seen in our study with spirometry values. Our prediction model has provided useful hypothesis generating results but requires validation in another similarly sized cohort which is difficult to source.

In conclusion, our study has demonstrated that exposure to traffic-related air pollutants, both perinatally and postnatally, have detrimental association with %FVC especially for those children born extremely preterm (<28 week's gestation). In our adjusted regression models based on current pollution exposure, we have shown that there may be up to 5.9% and 7.4% differences in %FVC between highest and lowest current pollution exposures for PM_2.5_ and NO_2,_ respectively. These data highlight the importance of air quality strategies to reduce any further detrimental impact on lung growth and function in this group of individuals with likely aberrant lung development, and who are already at heightened risk of long-term respiratory morbidity.^17^

## Data Availability

Data are available on reasonable request. All data relevant to the study are included in the article or uploaded as online supplemental information. Data from the RHiNO study are available to research collaborators subject to confidentiality and non-disclosure agreements. Contact Professor SK (kotechas@cardiff.ac.uk) for any data requests.
