# Supplementary material for: Impact of ambient air pollution on lung function in preterm-born school-aged children
Source: Thorax. 2024 Feb 15;79(6):553–63. doi: 10.1136/thorax-2023-220233 (PMC11137460; doi:10.1136/thorax-2023-220233)
Supplement: Supplementary data [file thorax-2023-220233supp001.pdf]

The impact of ambient air pollution on lung function in preterm-born school-aged children – Online Supplement

W John Watkins*, Christopher W Course*, Michael Cousins, Kylie Hart, Sarah J Kotecha, Sailesh Kotecha.

(*Joint 1st authors)

Department of Child Health, Cardiff University School of Medicine, Cardiff, United Kingdom

Corresponding Author: Professor Sailesh Kotecha

Department of Child Health

Cardiff University School of Medicine

Cardiff CF14 4XN

United Kingdom

kotechas@cardiff.ac.uk

Watkins WJ, *et al. Thorax* 2024; 79:553–563. doi: 10.1136/thorax-2023-220233

Supplementary Table 1: Mean and 95% confidence interval of lung function measures by demographic characteristics of cohort.

Variable		% Predicted FEV_1 Mean (95% CI)	% Predicted FVC Mean (95% CI)	Best FEV_1/FVC ratio Mean (95% CI)	% Predicted $FEF_{25-75\%}$ Mean (95% CI)
Gestation Band (weeks)	23-28	86.1 (83.7,88.6)	91.5 (89.4,93.6)	0.83 (0.81,0.84)	69.1 (65.0,73.1)
	29-31	89.9 (88.0,91.8)	93.4 (91.9,95.0)	0.84 (0.83,0.85)	74.9 (71.6,78.2)
	32-34	93.6 (92.1,95.0)	95.8 (94.3,97.2)	0.86 (0.85,0.87)	80.8 (78.6,83.1)
	Total	91.1 (90.0,92.2)	94.3 (93.3,95.2)	0.85 (0.84,0.85)	76.9 (75.1,78.6)
Sex	Male	90.6 (89.2,91.9)	93.7 (92.5,95.0)	0.83 (0.83,0.84)	77.5 (75.1,79.8)
	Female	91.7 (90.0,93.4)	94.8 (93.4,96.3)	0.86 (0.85,0.87)	76.2 (73.7,78.8)
IUGR	No IUGR	91.6 (90.5,92.7)	94.6 (93.6,95.6)	0.85 (0.84,0.85)	77.8 (76.0,79.6)
	IUGR	86.8 (82.8,90.8)	91.8 (88.7,94.9)	0.83 (0.81,0.86)	69.1 (63.6,74.5)
Maternal Age	≤19	83.6 (76.7,90.5)	91.5 (85.4,97.5)	0.80 (0.76,0.83)	63.5 (54.5,72.5)
	20-24	89.0 (85.9,92.1)	92.1 (89.5,94.7)	0.85 (0.82,0.87)	76.0 (70.9,81.2)
	25-29	91.3 (89.3,93.4)	94.7 (92.7,96.6)	0.85 (0.83,0.86)	77.1 (73.6,80.6)
	30-34	93.3 (91.3,95.3)	95.5 (93.8,97.8)	0.85 (0.84,0.86)	79.8 (76.7,82.9)
	35-39	90.9 (88.9,92.9)	94.3 (92.3,96.4)	0.85 (0.83,0.86)	75.8 (72.3,79.2)
	≥40	88.9 (83.4,94.3)	92.4 (88.3,96.6)	0.84 (0.80,0.88)	75.8 (67.1,84.6)
Broncho-pulmonary Dysplasia (BPD)	No BPD	92.3 (91.2,93.5)	94.8 (93.8,95.9)	0.85 (0.85,0.86)	78.9 (77.0,80.8)
	BPD	86.2 (83.7,88.8)	92.0 (89.8,94.1)	0.82 (0.8,0.84)	68.7 (64.6,72.8)
Antenatal smoking	No	91.1 (89.9,92.3)	94.2 (93.2,95.3)	0.85 (0.84,0.85)	76.9 (75.1,78.8)
	Yes	90.7 (87.8,93.7)	93.7 (90.9,96.4)	0.85 (0.83,0.87)	76.9 (71.2,82.6)
Postnatal smoking	No	91.2 (90.0,92.4)	94.2 (93.2,95.3)	0.85 (0.84,0.85)	77.3 (75.4,79.1)
	Yes	90.3 (87.7,92.9)	94.1 (91.8,96.4)	0.84 (0.82,0.86)	74.5 (69.8,79.3)
WIMD 2014 Quintile Birth	1 – least	91.9 (89.0,94.7)	94.3 (91.9,96.8)	0.85 (0.84,0.87)	78.9 (74.1,83.7)
	2	91.6 (89.5,93.7)	94.7 (92.7,96.8)	0.85 (0.83,0.86)	77.7 (74.0,81.3)
	3	91.3 (88.9,93.7)	95.2 (93.1,97.3)	0.84 (0.82,0.85)	75.9 (72.0,79.8)
	4	90.0 (86.8,93.2)	94.3 (91.5,97.0)	0.83 (0.82,0.85)	73.4 (68.8,78.0)
	5 – most	90.7 (88.7,92.6)	93.4 (91.6,95.3)	0.85 (0.84,0.86)	76.7 (73.5,79.9)
WIMD 2014 Quintile Time of Spirometry	1 – least	91.1 (88.4,93.8)	93.2 (90.7,95.7)	0.86 (0.84,0.87)	78.8 (74.1,83.4)
	2	92.2 (89.9,94.6)	96.1 (93.9,98.3)	0.84 (0.82,0.86)	76.7 (72.8,80.5)
	3	89.9 (87.4,92.3)	94.0 (91.9,96.1)	0.84 (0.82,0.85)	74.8 (70.8,78.8)
	4	92.5 (89.6,95.4)	94.3 (91.7,96.8)	0.86 (0.84,0.87)	80.6 (76.0,85.2)
	5 – most	90.6 (88.7,92.5)	94.0 (92.2,95.7)	0.84 (0.83,0.86)	75.5 (72.4,78.5)

WIMD: Welsh Index of Multiple Deprivation; IUGR: Intrauterine Growth Restriction. %FEV1: Percent predicted forced expiratory volume in 1 second; %FVC: Percent predicted forced vital capacity; FEV1/FVC ratio: ratio of percent predicted forced expiratory volume in 1 second to forced vital capacity; %FEF25-75%: Forced Expiratory Flow at 25-75% of forced vital capacity; 95% CI: 95% confidence interval.

Watkins WJ, *et al. Thorax* 2024; 79:553–563. doi: 10.1136/thorax-2023-220233

Supplementary Table 2: Mean and 95% confidence interval of annual pollutant measures by demographic characteristics of cohort.

Variable		$PM_{2.5}$ Birth Mean (95% CI)	PM_{10} Birth Mean (95% CI)	NO_2 Birth Mean (95% CI)	SO_2 Birth Mean (95% CI)
Gestation Band (weeks)	23-28	9.87 (9.61,10.13)	15.54 (15.11,15.96)	15.95 (14.87,17.04)	3.08 (2.68,3.49)
	29-31	9.6 (9.4,9.79)	15.13 (14.83,15.43)	15.1 (14.16,16.03)	2.8 (2.55,3.06)
	32-34	9.74 (9.61,9.88)	15.37 (15.14,15.6)	15.67 (14.98,16.36)	3.05 (2.87,3.23)
	Total	9.72 (9.62,9.83)	15.33 (15.16,15.5)	15.55 (15.06,16.04)	2.98 (2.84,3.12)
Sex	Male	9.67 (9.53,9.81)	15.24 (15.01,15.47)	15.37 (14.7,16.04)	2.93 (2.73,3.12)
	Female	9.78 (9.63,9.93)	15.42 (15.18,15.67)	15.74 (15.02,16.47)	3.04 (2.83,3.25)
IUGR	No IUGR	9.69 (9.59,9.8)	15.32 (15.14,15.5)	15.41 (14.9,15.92)	2.96 (2.81,3.11)
	IUGR	9.96 (9.63,10.29)	15.42 (14.88,15.95)	16.73 (15.02,18.43)	3.16 (2.71,3.61)
Maternal Age	≤19	9.82 (9.3,10.35)	15.38 (14.64,16.11)	16.61 (14.12,19.09)	3.21 (2.65,3.76)
	20-24	9.79 (9.49,10.08)	15.55 (15.06,16.05)	15.61 (14.44,16.77)	3.3 (2.86,3.74)
	25-29	9.79 (9.59,9.98)	15.31 (14.97,15.65)	15.73 (14.71,16.75)	3.46 (3.06,3.86)
	30-34	9.67 (9.46,9.87)	15.3 (14.98,15.61)	15.34 (14.43,16.26)	2.69 (2.49,2.89)
	35-39	9.68 (9.48,9.88)	15.18 (14.83,15.53)	15.62 (14.53,16.7)	2.77 (2.55,2.99)
	≥40	9.72 (9.23,10.2)	15.68 (15.04,16.32)	14.79 (12.55,17.03)	2.53 (2.1,2.96)
Broncho-pulmonary Dysplasia (BPD)	No BPD	9.66 (9.55,9.77)	15.25 (15.06,15.43)	15.37 (14.82,15.91)	3 (2.84,3.16)
	BPD	9.97 (9.7,10.24)	15.65 (15.25,16.06)	16.29 (15.13,17.44)	2.9 (2.6,3.2)
Antenatal smoking	No	9.72 (9.61,9.83)	15.29 (15.11,15.47)	15.47 (14.94,16.01)	2.96 (2.8,3.12)
	Yes	9.79 (9.45,10.12)	15.65 (15.15,16.14)	16.43 (14.97,17.89)	3.18 (2.79,3.57)
Postnatal smoking	No	9.7 (9.59,9.82)	15.29 (15.11,15.47)	15.39 (14.85,15.92)	2.94 (2.79,3.09)
	Yes	9.82 (9.55,10.1)	15.52 (15.07,15.97)	16.57 (15.28,17.86)	3.26 (2.88,3.65)
WIMD 2014 Quintile Birth	1 – least	9.8 (9.58,10.02)	15.58 (15.21,15.94)	16.66 (15.53,17.79)	3.06 (2.79,3.34)
	2	9.59 (9.38,9.8)	15.1 (14.75,15.45)	15 (13.94,16.07)	3.18 (2.75,3.61)
	3	9.55 (9.29,9.81)	15.03 (14.65,15.42)	14.04 (12.94,15.13)	2.96 (2.62,3.3)
	4	9.65 (9.35,9.94)	15.25 (14.78,15.72)	15.2 (13.77,16.63)	2.97 (2.62,3.32)
	5 – most	9.94 (9.74,10.13)	15.59 (15.26,15.92)	16.5 (15.59,17.41)	2.8 (2.59,3.02)
WIMD 2014 Quintile Time of spirometry	1 – least	9.92 (9.71,10.13)	15.63 (15.26,16)	16.8 (15.66,17.94)	3.23 (2.92,3.54)
	2	9.56 (9.31,9.8)	15.17 (14.76,15.59)	15.13 (13.91,16.34)	3.21 (2.73,3.7)
	3	9.49 (9.26,9.71)	15.03 (14.69,15.36)	14.09 (13.1,15.09)	2.9 (2.62,3.18)
	4	9.4 (9.13,9.68)	14.87 (14.42,15.32)	14.34 (12.96,15.71)	2.69 (2.35,3.04)
	5 – most	10.05 (9.86,10.24)	15.71 (15.39,16.03)	16.79 (15.89,17.68)	2.92 (2.69,3.14)

WIMD: Welsh Index of Multiple Deprivation; IUGR: Intrauterine Growth Restriction. %FEV1: Percent predicted forced expiratory volume in 1 second; %FVC: Percent predicted forced vital capacity; $PM_{2.5}$: Particulate matter with a diameter of 2.5μm; PM_{10}: Particulate matter with a diameter of 10μm; NO_2: Nitrogen dioxide; SO_2: Sulphur dioxide; 95% CI: 95% confidence interval

Watkins WJ, *et al. Thorax* 2024; 79:553–563. doi: 10.1136/thorax-2023-220233

Supplementary Figure 1: Directed Acyclic Graph (DAG) showing relationships between gestational age and pollution and lung function with important, potentially confounding variables.

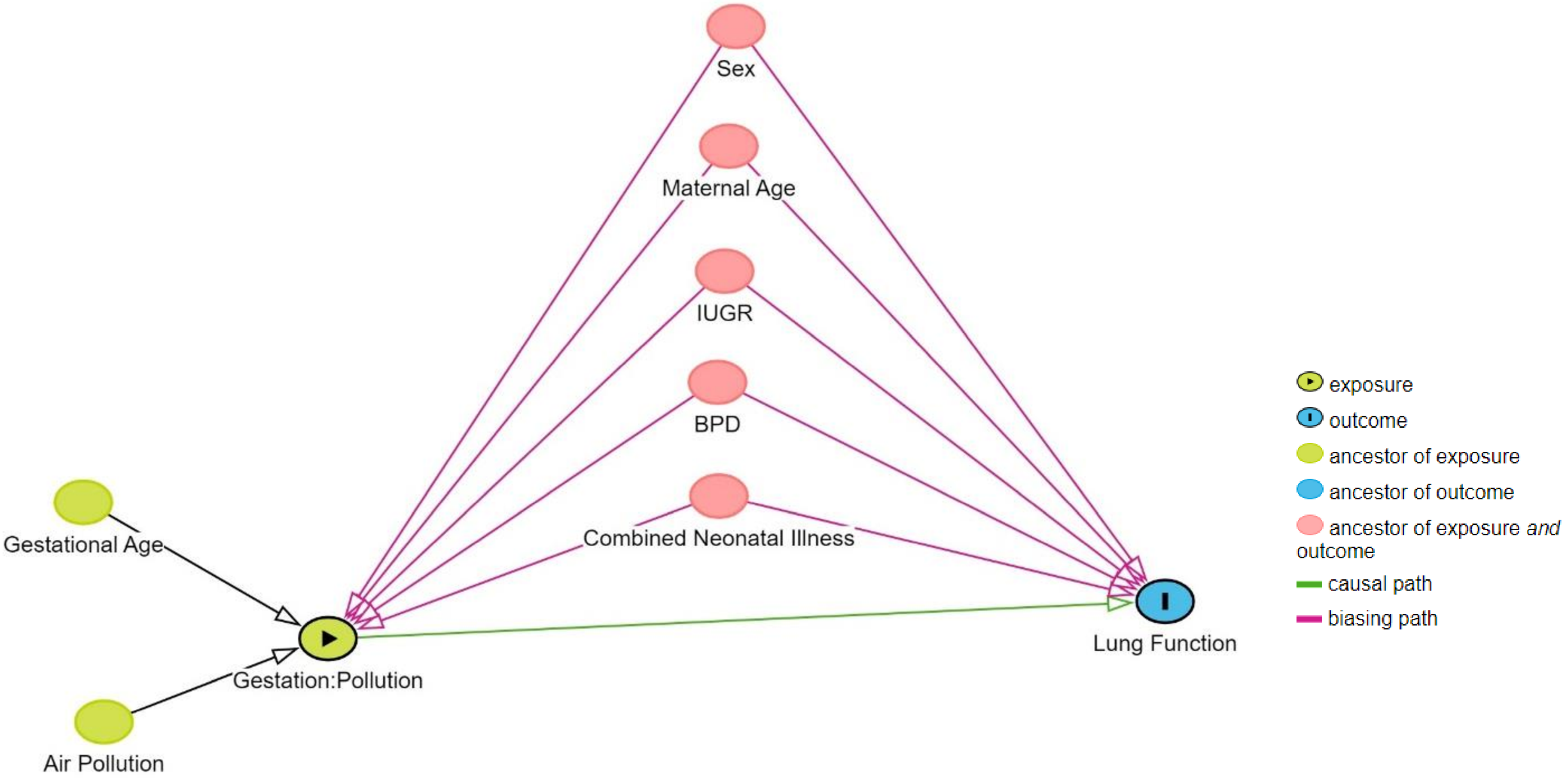

BPD: Bronchopulmonary dysplasia; IUGR: Intrauterine Growth Restriction.; Combined Neonatal Illness: Presence of one or more of following: necrotising enterocolitis, intraventricular haemorrhage, retinopathy of prematurity, and/or patent ductus arteriosus.

Supplementary Table 3: Adjusted linear regression models between pollutants and lung function measures by gestational age group

Spirometry Measure	Gestational Group (weeks)	Birth			
		**$PM_{2.5}$**	**PM_{10}**	**NO_2**	**SO_2**
		**β (95% CI) [p-value]**	**β (95% CI) [p-value]**	**β (95% CI) [p-value]**	**β (95% CI) [p-value]**
$\%FEV_1$	23-28	-0.34 (-1.28,0.61) [0.48]	-0.46 (-1.04,0.12) [0.11]	-0.09 (-0.36,0.19) [0.54]	-0.15 (-1.07,0.77) [0.74]
	29-31	-0.23 (-1.13,0.67) [0.62]	-0.40 (-0.95,0.16) [0.16]	-0.03 (-0.23,0.18) [0.81]	-0.06 (-0.90,0.77) [0.88]
	32-34	-0.04 (-0.92,0.84) [0.92]	-0.27 (-0.82,0.27) [0.32]	0.07 (-0.12,0.26) [0.48]	0.22 (-0.55,0.98) [0.58]
%FVC	23-28	-0.57 (-1.45,0.31) [0.20]	-0.66 (-1.20,-0.13) [0.016]	-0.16 (-0.42,0.09) [0.22]	-0.08 (-0.94,0.78) [0.86]
	29-31	-0.44 (-1.28,0.40) [0.30]	-0.58 (-1.1,-0.07) [0.027]	-0.07 (-0.27,0.12) [0.46]	0.04 (-0.74,0.82) [0.92]
	32-34	-0.31 (-1.13,0.52) [0.46]	-0.49 (-1.0,0.02) [0.058]	-0.01 (-0.20,0.17) [0.88]	0.22 (-0.50,0.93) [0.55]
$\%FEV_1/FVC$ ratio	23-28	0.002 (-0.004,0.01) [0.56]	0 (-0.004,0.004) [0.99]	0.001 (-0.001,0.002) [0.47]	-0.001 (-0.01,0.005) [0.71]
	29-31	0.001 (-0.004,0.01) [0.63]	0 (-0.004,0.003) [0.88]	0 (-0.001,0.002) [0.65]	-0.002 (-0.01,0.003) [0.50]
	32-34	0.002 (-0.003,0.01) [0.43]	0 (-0.003,0.004) [0.86]	0.001 (0,0.002) [0.18]	0 (-0.005,0.005) [0.99]
$\%FEF_{25-75\%}$	23-28	-0.09 (-1.62,1.44) [0.90]	-0.09 (-1.03,0.85) [0.85]	-0.02 (-0.46,0.43) [0.94]	-0.48 (-1.97,1.01) [0.53]
	29-31	-0.09 (-1.56,1.37) [0.89]	-0.08 (-0.99,0.8) [0.85]	-0.02 (-0.36,0.32) [0.92]	-0.404 (-1.76,0.95) [0.56]
	32-34	0.24 (-1.19,1.67) [0.74]	0.13 (-0.76,1.01) [0.78]	0.17 (-0.14,0.49) [0.28]	0.17 (-1.07,1.40) [0.79]
		**Current**			
		**$PM_{2.5}$**	**PM_{10}**	**NO_2**	**SO_2**
		**β (95% CI) [p-value]**	**β (95% CI) [p-value]**	**β (95% CI) [p-value]**	**β (95% CI) [p-value]**
$\%FEV_1$	23-28	-0.74 (-1.89, 0.42) [0.21]	-0.38 (-1.20,0.44) [0.37]	-0.21 (-0.53,0.11) [0.19]	-0.78 (-3.07,1.52) [0.51]
	29-31	-0.53 (-1.63,0.57) [0.35]	-0.24 (-1.02,0.55) [0.55]	-0.12 (-0.36,0.12) [0.31]	-0.19 (-1.77,1.39) [0.82]
	32-34	-0.28 (-1.36,0.80) [0.61]	-0.08 (-0.85,0.69) [0.84]	-0.02 (-0.24,0.21) [0.89]	0.40 (-1.06,1.87) [0.59]
%FVC	23-28	-1.19 (-2.26,-0.11) [0.031]	-0.73 (-1.50,0.04) [0.06]	-0.29 (-0.59,0.004) [0.05]	-1.35 (-3.49,0.79) [0.22]
	29-31	-0.97 (-1.99,0.06) [0.06]	-0.58 (-1.31,0.15) [0.12]	-0.20 (-0.42,0.03) [0.09]	-0.57 (-2.04,0.90) [0.45]
	32-34	-0.79 (-1.79,0.21) [0.12]	-0.47 (-1.19,0.25) [0.20]	-0.13 (-0.34,0.07) [0.21]	-0.30 (-1.67,1.07) [0.67]
$\%FEV_1/FVC$ ratio	23-28	0.003 (-0.004,0.01) [0.41]	0.003 (-0.002,0.01) [0.31]	0 (-0.002,0.002) [0.66]	0.01 (-0.01,0.02) [0.50]
	29-31	0.003 (-0.004,0.01) [0.41]	0.003 (-0.002,0.01) [0.31]	0 (-0.001,0.002) [0.57]	0.003 (-0.01,0.01) [0.54]
	32-34	0.004 (-0.003,0.011) [0.25]	0.003 (-0.002,0.01) [0.19]	0.001 (0,0.002) [0.13]	0.01 (-0.002,0.02) [0.11]
$\%FEF_{25-75\%}$	23-28	-0.002 (-1.90,1.90) [0.99]	0.15 (-1.2,1.51) [0.82]	-0.08 (-0.61,0.44) [0.76]	0.26 (-3.51,4.03) [0.89]
	29-31	0.11 (-1.70,1.92) [0.91]	0.23 (-1.07,1.52) [0.73]	-0.07 (-0.46,0.32) [0.73]	0.27 (-2.33,2.86) [0.84]
	32-34	0.56 (-1.21,2.33) [0.53]	0.52 (-0.75,1.79) [0.42]	0.16 (-0.20,0.52) [0.39]	1.72 (-0.69,4.13) [0.16]

Beta value (95% confidence interval) given for modelling for each spirometry variable and pollutant by gestational age band adjusted for Sex, IUGR, BPD, combined illness and maternal age.
$\%FEV_1$: Percent predicted forced expiratory volume in 1 second; %FVC: Percent predicted forced vital capacity; FEV_1/FVC ratio: ratio of percent predicted forced expiratory volume in 1 second to forced vital capacity; $\%FEF_{25-75\%}$: Forced Expiratory Flow at 25-75% of forced vital capacity;
$PM_{2.5}$: Particulate matter with a diameter of 2.5μm; PM_{10}: Particulate matter with a diameter of 10μm; NO_2: Nitrogen dioxide; SO_2: Sulphur dioxide; 95% CI: 95% confidence interval.

Supplementary Table 4: Univariable analysis with the average value of the pollutant between birth and time of spirometry by lung function measure.

%FVC

Pollutant	BETA	SE	P value
PM_{10}	-0.085	0.43	0.85
$PM_{2.5}$	0.11	0.61	0.85
NO_2	-0.038	0.12	0.75
SO_2	0.13	0.81	0.87

%FEV$_1$

Pollutant	BETA	SE	P value
PM_{10}	0.1018	0.5017	0.839
$PM_{2.5}$	0.274	0.7007	0.696
NO_2	0.0088	0.1366	0.948
SO_2	0.6093	0.9359	0.516

%FEF$_{25-75}$

Pollutant	BETA	SE	P value
PM_{10}	0.6829	0.8149	0.403
$PM_{2.5}$	0.7445	1.1388	0.514
NO_2	0.1010	0.2221	0.650
SO_2	1.853	1.519	0.224

FEV$_1$/FVC

Pollutant	BETA	SE	P value
PM_{10}	0.0008771	0.0029981	0.77
$PM_{2.5}$	0.001135	0.004188	0.787
NO_2	0.0003484	0.0008162	0.67
SO_2	0.003978	0.005592	0.477

%FEV$_1$: Percent predicted forced expiratory volume in 1 second; %FVC: Percent predicted forced vital capacity; FEV$_1$/FVC ratio: ratio of percent predicted forced expiratory volume in 1 second to forced vital capacity; %FEF$_{25-75\%}$: Forced Expiratory Flow at 25-75% of forced vital capacity; $PM_{2.5}$: Particulate matter with a diameter of 2.5µm; PM_{10}: Particulate matter with a diameter of 10µm; NO_2: Nitrogen dioxide; SO_2: Sulphur dioxide; SE: Standard error.

Supplementary Table 5: Predictive Models for FEV_1 over range of pollutant exposure at birth and time of spirometry

Variable		For minimum pollutant exposure	For mean pollutant exposure	For maximum pollutant exposure	Min-to-Max Difference (% of mean)	Beta (95% CI)	p-value
**Birth $PM_{2.5}$ (μg/m^3)**		6.9	9.72	13.31	6.41 (65.94)		
**%FEV_1 by Gestation (weeks)**	23-28	87.06	86.11	84.91	2.15 (2.5)	-0.34 (-1.28,0.6)	0.48
	29-31	90.57	89.93	89.11	1.46 (1.62)	-0.23 (-1.13,0.67)	0.62
	32-34	93.69	93.57	93.42	0.26 (0.28)	-0.04 (-0.92,0.84)	0.93
**Birth PM_{10} (μg/m^3)**		10.1	15.33	20.56	10.46 (68.26)		
**%FEV_1 by Gestation (weeks)**	23-28	88.52	86.11	83.7	4.83 (5.6)	-0.46 (-1.04,0.12)	0.12
	29-31	92	89.93	87.85	4.15 (4.61)	-0.4 (-0.95,0.16)	0.16
	32-34	94.99	93.57	92.15	2.85 (3.04)	-0.27 (-0.82,0.27)	0.33
**Birth SO_2 (μg/m^3)**		0.81	2.98	15.89	15.08 (505.82)		
**%FEV_1 by Gestation (weeks)**	23-28	86.44	86.11	84.13	2.31 (2.68)	-0.15 (-1.07,0.77)	0.74
	29-31	90.07	89.93	89.11	0.96 (1.07)	-0.06 (-0.9,0.77)	0.88
	32-34	93.1	93.57	96.37	-3.27 (-3.49)	0.22 (-0.55,0.98)	0.58
**Birth NO_2 (μg/m^3)**		4.48	15.55	34.93	30.45 (195.81)		
**%FEV_1 by Gestation (weeks)**	23-28	87.05	86.11	84.46	2.59 (3.01)	-0.09 (-0.36,0.19)	0.54
	29-31	90.21	89.93	89.44	0.77 (0.85)	-0.03 (-0.23,0.18)	0.81
	32-34	92.8	93.57	94.93	-2.13 (-2.27)	0.07 (-0.12,0.26)	0.48
**Current $PM_{2.5}$ (μg/m^3)**		5.58	7.99	10.58	5.01 (62.66)		
**%FEV_1 by Gestation (weeks)**	23-28	87.89	86.11	84.2	3.69 (4.29)	-0.74 (-1.89,0.42)	0.21
	29-31	91.2	89.93	88.56	2.64 (2.94)	-0.53 (-1.63,0.57)	0.35
	32-34	94.25	93.57	92.85	1.4 (1.5)	-0.28 (-1.36,0.8)	0.61
**Current PM_{10} (μg/m^3)**		8.83	12.5	16.31	7.48 (59.84)		
**%FEV_1 by Gestation (weeks)**	23-28	87.5	86.11	84.67	2.83 (3.29)	-0.38 (-1.2,0.44)	0.37
	29-31	90.8	89.93	89.02	1.78 (1.98)	-0.24 (-1.02,0.55)	0.55
	32-34	93.87	93.57	93.27	0.6 (0.64)	-0.08 (-0.85,0.69)	0.84
**Current SO_2 (μg/m^3)**		0.55	2.07	8.82	8.27 (400.36)		
**%FEV_1 by Gestation (weeks)**	23-28	87.29	86.11	80.86	6.43 (7.47)	-0.78 (-3.07,1.52)	0.51
	29-31	90.21	89.93	88.66	1.55 (1.73)	-0.19 (-1.77,1.39)	0.82
	32-34	92.96	93.57	96.28	-3.32 (-3.55)	0.4 (-1.06,1.87)	0.59

Watkins WJ, et al. Thorax 2024; 79:553–563. doi: 10.1136/thorax-2023-220233

**Current NO_2 (μg/m^3)**		3.8	13.35	29.24	25.44 (190.6)		
**%FEV_1 by Gestation (weeks)**	23-28	88.15	86.11	82.72	5.42 (6.3)	-0.21 (-0.53,0.11)	0.19
	29-31	91.09	89.93	87.99	3.11 (3.45)	-0.12 (-0.36,0.12)	0.31
	32-34	93.72	93.57	93.33	0.39 (0.41)	-0.02 (-0.24,0.2)	0.89

%FEV1: Percent predicted forced expiratory volume in 1 second; $PM_{2.5}$: Particulate matter with a diameter of 2.5μm; PM_{10}: Particulate matter with a diameter of 10μm; NO_2: Nitrogen dioxide; SO_2: Sulphur dioxide; 95% CI: 95% confidence interval.

Watkins WJ, *et al. Thorax* 2024; 79:553–563. doi: 10.1136/thorax-2023-220233

Supplementary Table 6: Predictive Models for FEV_1/FVC ratio over range of pollutant exposure at birth and time of spirometry

Variable		For minimum pollutant exposure	For mean pollutant exposure	For maximum pollutant exposure	Min-to-Max Difference (% of mean)	Beta (95% CI)	p-value
**Birth $PM_{2.5}$ (μg/m^3)**		6.9	9.72	13.31	6.41 (65.94)		
**FEV_1/FVC by Gestation (weeks)**	23-28	0.82	0.83	0.83	-0.01 (-1.32)	0 (0,0.01)	0.57
	29-31	0.84	0.84	0.84	-0.01 (-1.03)	0 (0,0.01)	0.63
	32-34	0.85	0.86	0.86	-0.01 (-1.62)	0 (0,0.01)	0.44
**Birth PM_{10} (μg/m^3)**		10.1	15.33	20.56	10.46 (68.26)		
**FEV_1/FVC by Gestation (weeks)**	23-28	0.83	0.83	0.83	0 (0)	0 (0,0)	1
	29-31	0.84	0.84	0.84	0 (0.31)	0 (0,0)	0.89
	32-34	0.86	0.86	0.86	0 (-0.36)	0 (0,0)	0.87
**Birth SO_2 (μg/m^3)**		0.81	2.98	15.89	15.08 (505.82)		
**FEV_1/FVC by Gestation (weeks)**	23-28	0.83	0.83	0.81	0.02 (1.96)	0 (-0.01,0)	0.71
	29-31	0.84	0.84	0.82	0.03 (3.21)	0 (-0.01,0)	0.5
	32-34	0.86	0.86	0.86	0 (0.07)	0 (0,0)	0.99
**Birth NO_2 (μg/m^3)**		4.48	15.55	34.93	30.45 (195.81)		
**FEV_1/FVC by Gestation (weeks)**	23-28	0.82	0.83	0.84	-0.02 (-2.32)	0 (0,0)	0.46
	29-31	0.84	0.84	0.85	-0.01 (-1.09)	0 (0,0)	0.65
	32-34	0.85	0.86	0.87	-0.02 (-2.89)	0 (0,0)	0.18
**Current $PM_{2.5}$ (μg/m^3)**		5.58	7.99	10.58	5.01 (62.66)		
**FEV_1/FVC by Gestation (weeks)**	23-28	0.82	0.83	0.83	-0.02 (-1.83)	0 (0,0.01)	0.41
	29-31	0.83	0.84	0.85	-0.01 (-1.71)	0 (0,0.01)	0.41
	32-34	0.85	0.86	0.87	-0.02 (-2.31)	0 (0,0.01)	0.25
**Current PM_{10} (μg/m^3)**		8.83	12.5	16.31	7.48 (59.84)		
**FEV_1/FVC by Gestation (weeks)**	23-28	0.82	0.83	0.84	-0.02 (-2.38)	0 (0,0.01)	0.31
	29-31	0.83	0.84	0.85	-0.02 (-2.23)	0 (0,0.01)	0.31
	32-34	0.85	0.86	0.87	-0.02 (-2.8)	0 (0,0.01)	0.19
**Current SO_2 (μg/m^3)**		0.55	2.07	8.82	8.27 (400.36)		
**FEV_1/FVC by Gestation (weeks)**	23-28	0.82	0.83	0.86	-0.04 (-4.91)	0 (-0.01,0.02)	0.5
	29-31	0.84	0.84	0.86	-0.03 (-2.99)	0 (-0.01,0.01)	0.54
	32-34	0.85	0.86	0.91	-0.06 (-7.13)	0.01 (0,0.02)	0.11

**Current NO_2 (μg/m^3)**		3.8	13.35	29.24	25.44 (190.6)		
**FEV_1/FVC by Gestation (weeks)**	23-28	0.82	0.83	0.83	-0.01 (-1.35)	0 (0,0)	0.66
	29-31	0.84	0.84	0.85	-0.01 (-1.28)	0 (0,0)	0.57
	32-34	0.85	0.86	0.87	-0.03 (-3.13)	0 (0,0)	0.13

FEV1/FVC ratio: ratio of percent predicted forced expiratory volume in 1 second to forced vital capacity; $PM_{2.5}$: Particulate matter with a diameter of 2.5μm; PM_{10}: Particulate matter with a diameter of 10μm; NO_2: Nitrogen dioxide; SO_2: Sulphur dioxide; 95% CI: 95% confidence interval.

Supplementary Table 7: Predictive Models for %$FEF_{25-75\%}$ over range of pollutant exposure at birth and time of spirometry

Variable		For minimum pollutant exposure	For mean pollutant exposure	For maximum pollutant exposure	Min-to-Max Difference (% of mean)	Beta (95% CI)	p-value
**Birth $PM_{2.5}$ (μg/m^3)**		6.9	9.72	13.31	6.41 (65.94)		
**%$FEF_{25-75\%}$ by Gestation (weeks)**	23-28	69.31	69.05	68.72	0.59 (0.85)	-0.09 (-1.62,1.43)	0.91
	29-31	75.15	74.88	74.55	0.6 (0.81)	-0.09 (-1.56,1.37)	0.9
	32-34	80.16	80.84	81.71	-1.55 (-1.92)	0.24 (-1.19,1.67)	0.74
**Birth PM_{10} (μg/m^3)**		10.1	15.33	20.56	10.46 (68.26)		
**%$FEF_{25-75\%}$ by Gestation (weeks)**	23-28	69.51	69.05	68.59	0.91 (1.32)	-0.09 (-1.03,0.85)	0.86
	29-31	75.32	74.88	74.45	0.87 (1.16)	-0.08 (-0.99,0.82)	0.86
	32-34	80.18	80.84	81.51	-1.33 (-1.64)	0.13 (-0.76,1.01)	0.78
**Birth SO_2 (μg/m^3)**		0.81	2.98	15.89	15.08 (505.82)		
**%$FEF_{25-75\%}$ by Gestation (weeks)**	23-28	70.09	69.05	62.86	7.23 (10.47)	-0.48 (-1.97,1.01)	0.53
	29-31	75.76	74.88	69.66	6.1 (8.15)	-0.4 (-1.76,0.95)	0.56
	32-34	80.48	80.84	82.98	-2.5 (-3.09)	0.17 (-1.07,1.4)	0.79
**Birth NO_2 (μg/m^3)**		4.48	15.55	34.93	30.45 (195.81)		
**%$FEF_{25-75\%}$ by Gestation (weeks)**	23-28	69.24	69.05	68.71	0.53 (0.77)	-0.02 (-0.46,0.42)	0.94
	29-31	75.08	74.88	74.54	0.55 (0.73)	-0.02 (-0.36,0.32)	0.92
	32-34	78.91	80.84	84.22	-5.3 (-6.56)	0.17 (-0.14,0.49)	0.28
**Current $PM_{2.5}$ (μg/m^3)**		5.58	7.99	10.58	5.01 (62.66)		
**%$FEF_{25-75\%}$ by Gestation (weeks)**	23-28	69.05	69.05	69.05	0.01 (0.01)	0 (-1.9,1.9)	1
	29-31	74.63	74.88	75.16	-0.54 (-0.71)	0.11 (-1.7,1.92)	0.91
	32-34	79.49	80.84	82.3	-2.81 (-3.48)	0.56 (-1.21,2.33)	0.53
**Current PM_{10} (μg/m^3)**		8.83	12.5	16.31	7.48 (59.84)		
**%$FEF_{25-75\%}$ by Gestation (weeks)**	23-28	68.49	69.05	69.64	-1.15 (-1.67)	0.15 (-1.2,1.51)	0.82
	29-31	74.06	74.88	75.74	-1.68 (-2.25)	0.22 (-1.07,1.52)	0.73
	32-34	78.94	80.84	82.81	-3.87 (-4.78)	0.52 (-0.75,1.79)	0.42
**Current SO_2 (μg/m^3)**		0.55	2.07	8.82	8.27 (400.36)		
**%$FEF_{25-75\%}$ by Gestation (weeks)**	23-28	68.66	69.05	70.78	-2.12 (-3.07)	0.26 (-3.51,4.03)	0.89
	29-31	74.48	74.88	76.69	-2.21 (-2.95)	0.27 (-2.33,2.86)	0.84

	32-34	78.23	80.84	92.45	-14.22 (-17.59)	1.72 (-0.69,4.13)	0.16
**Current NO_2 (µg/m^3)**		3.8	13.35	29.24	25.44 (190.6)		
**%$FEF_{25-75\%}$ by Gestation (weeks)**	23-28	69.85	69.05	67.72	2.12 (3.07)	-0.08 (-0.61,0.44)	0.75
	29-31	75.53	74.88	73.81	1.72 (2.3)	-0.07 (-0.46,0.32)	0.73
	32-34	79.31	80.84	83.39	-4.07 (-5.04)	0.16 (-0.2,0.52)	0.39

%FEF25-75%: Forced Expiratory Flow at 25-75% of forced vital capacity; $PM_{2.5}$: Particulate matter with a diameter of 2.5µm; PM_{10}: Particulate matter with a diameter of 10µm; NO_2: Nitrogen dioxide; SO_2: Sulphur dioxide; 95% CI: 95% confidence interval.

Watkins WJ, *et al. Thorax* 2024; 79:553–563. doi: 10.1136/thorax-2023-220233

Supplementary Figure 2a: Predicted %FEV_1 over range of pollutant exposure by decile at time of birth. Point represents mean value with bar indicating 95% confidence interval.

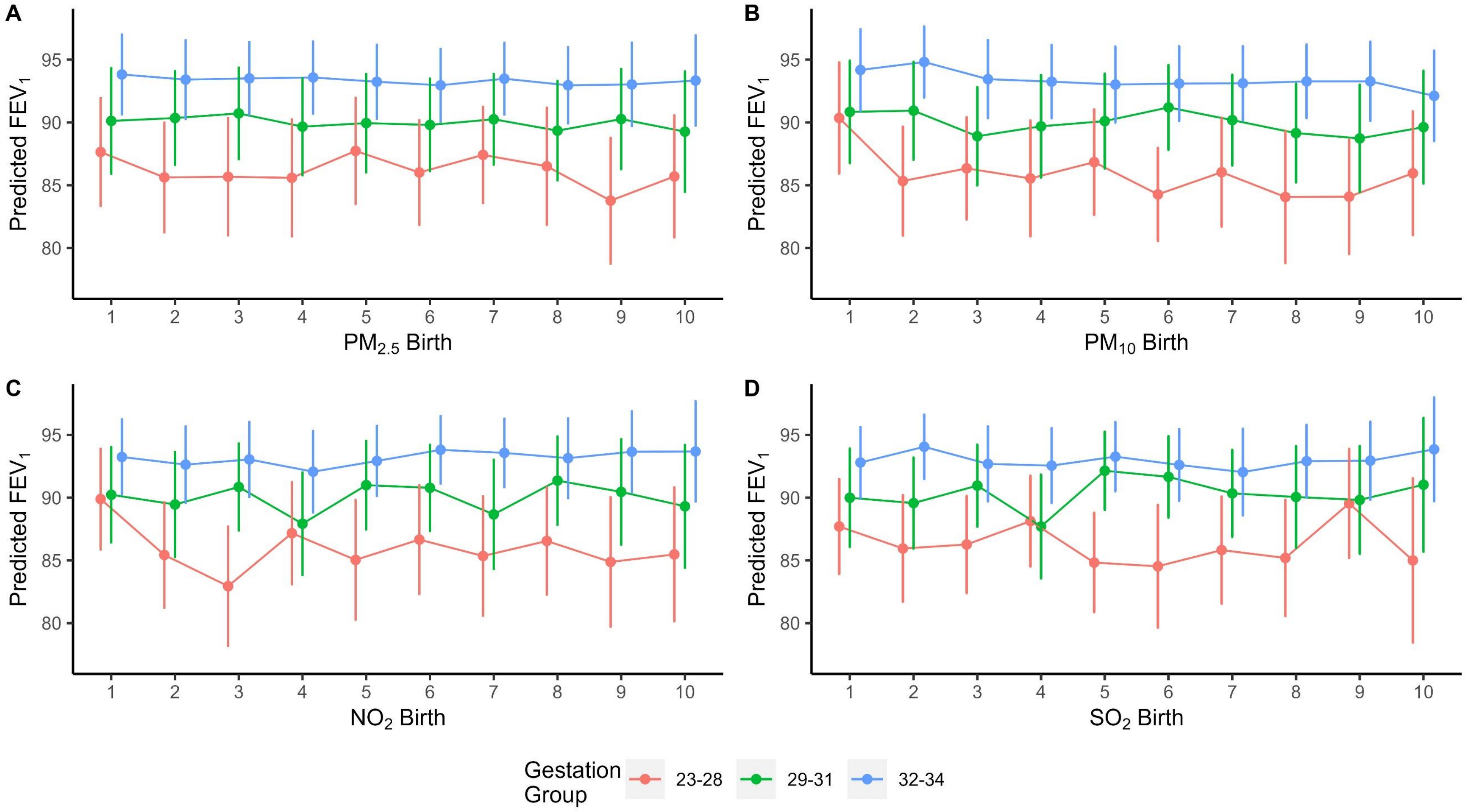

%FEV_1: Percentage predicted forced expiratory volume in 1 second; $PM_{2.5}$: Particulate matter with a diameter of 2.5µm; PM_{10}: Particulate matter with a diameter of 10µm; NO_2: Nitrogen dioxide; SO_2: Sulphur dioxide.

Supplementary Figure 2b: Predicted %FEV_1 over range of pollutant exposure by decile at time of spirometry. Point represents mean value with bar indicating 95% confidence interval.

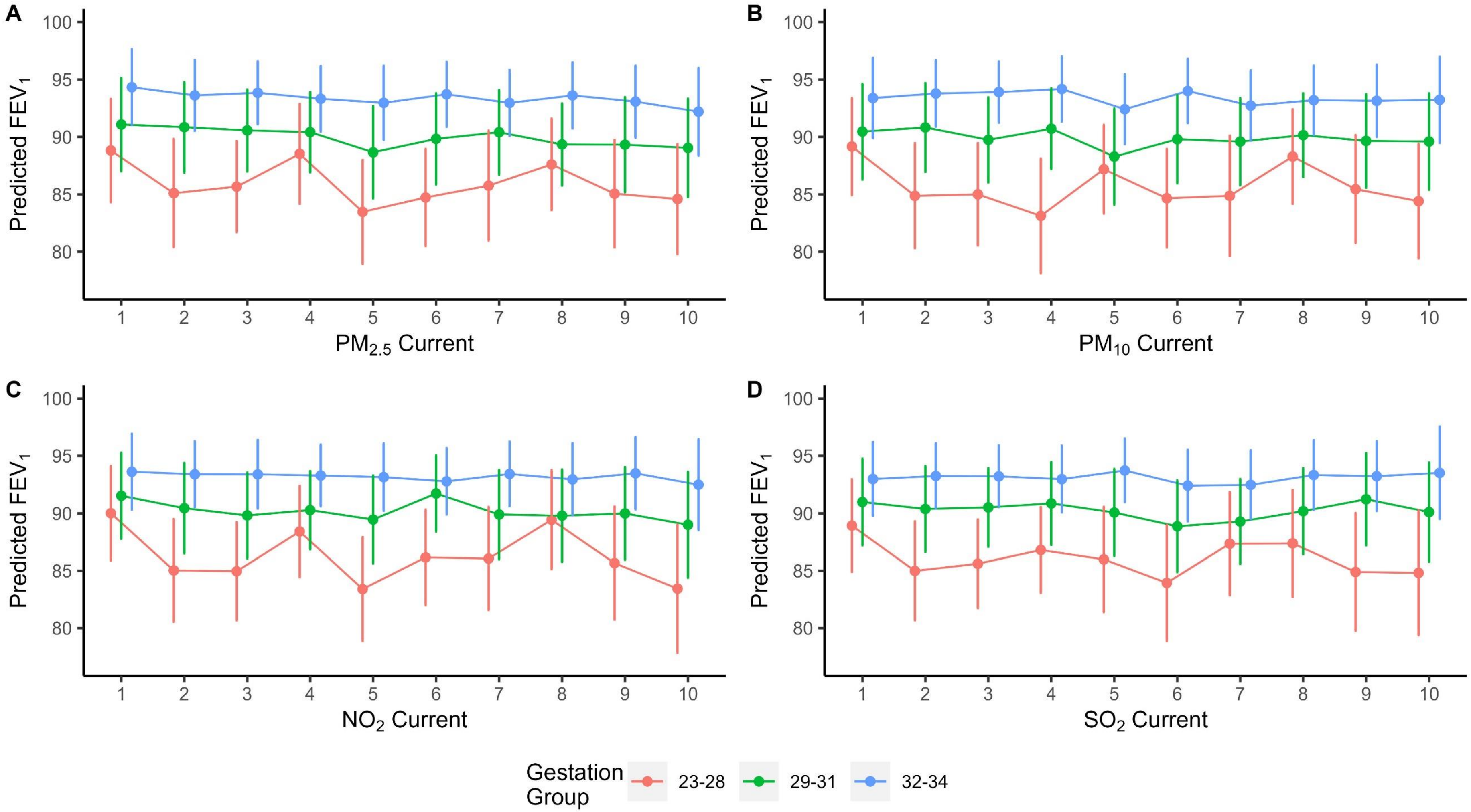

%FEV_1: Percentage predicted forced expiratory volume in 1 second; $PM_{2.5}$: Particulate matter with a diameter of 2.5µm; PM_{10}: Particulate matter with a diameter of 10µm; NO_2: Nitrogen dioxide; SO_2: Sulphur dioxide.

Watkins WJ, *et al. Thorax* 2024; 79:553–563. doi: 10.1136/thorax-2023-220233

Supplementary Figure 3a: Predicted FEV_1/FVC ratio over range of pollutant exposure by decile at time of birth. Point represents mean value with bar indicating 95% confidence interval.

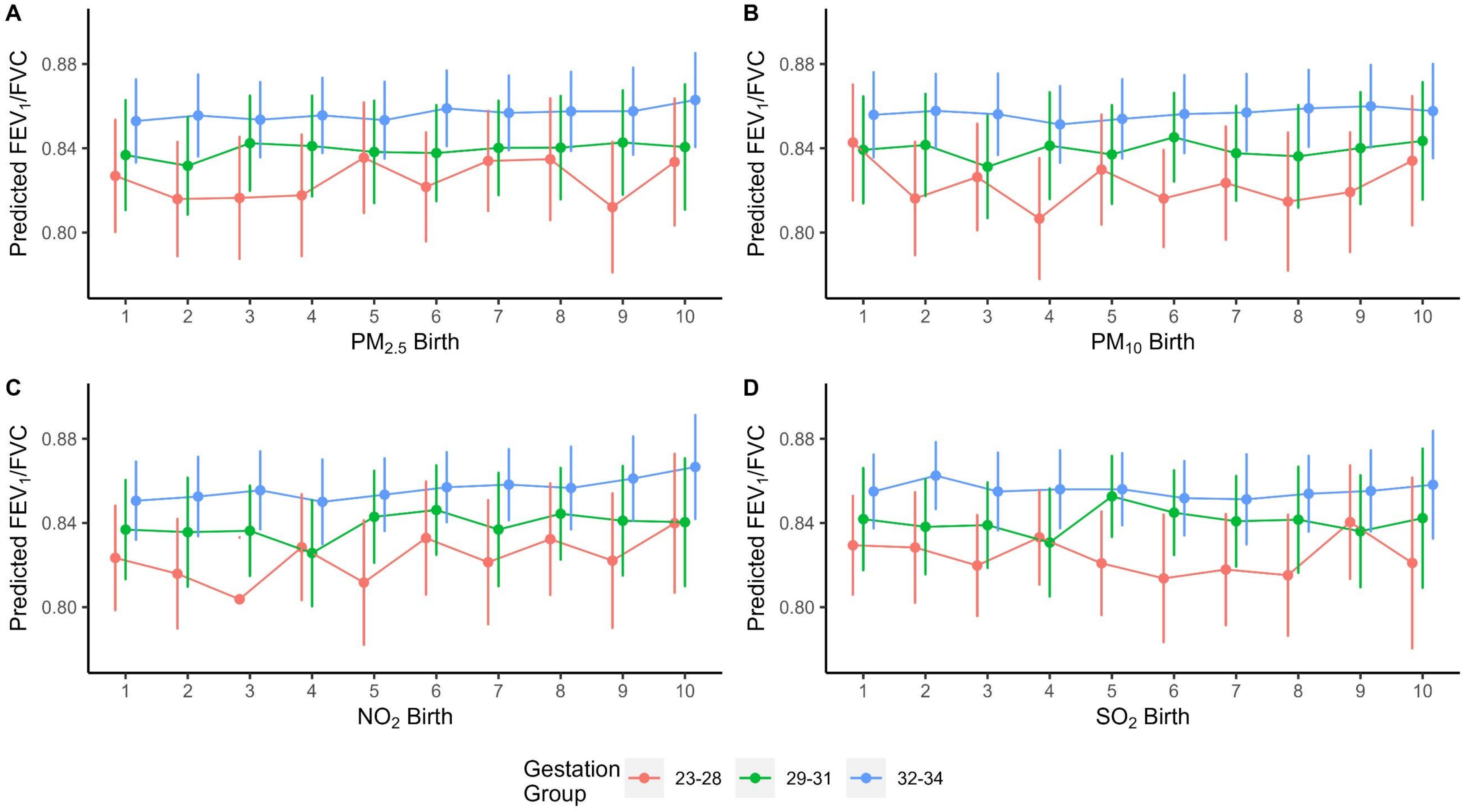

FEV_1/FVC: Percentage predicted forced expiratory volume in 1 second/ percent predicted forced vital capacity. $PM_{2.5}$: Particulate matter with a diameter of 2.5μm; PM_{10}: Particulate matter with a diameter of 10μm; NO_2: Nitrogen dioxide; SO_2: Sulphur dioxide.

Watkins WJ, *et al. Thorax* 2024; 79:553–563. doi: 10.1136/thorax-2023-220233

Supplementary Figure 3b: Predicted FEV_1/FVC ratio over range of pollutant exposure by decile at time of spirometry Point represents mean value with bar indicating 95% confidence interval.

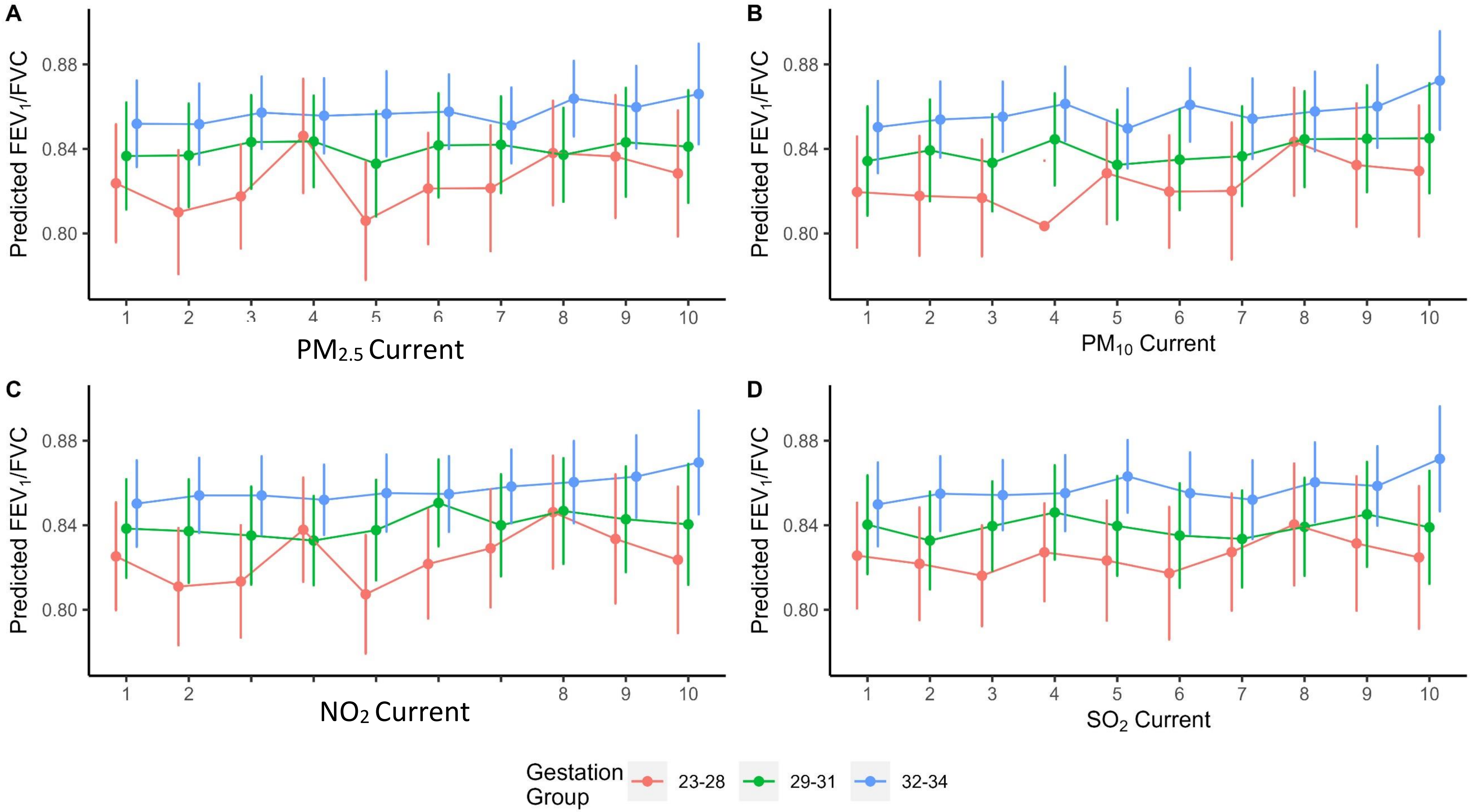

FEV_1/FVC: Percentage predicted forced expiratory volume in 1 second/ percent predicted forced vital capacity. $PM_{2.5}$: Particulate matter with a diameter of 2.5µm; PM_{10}: Particulate matter with a diameter of 10µm; NO_2: Nitrogen dioxide; SO_2: Sulphur dioxide.

Watkins WJ, *et al. Thorax* 2024; 79:553–563. doi: 10.1136/thorax-2023-220233

Supplementary Figure 4a: Predicted %$FEF_{25-75\%}$ over range of pollutant exposure by decile at time of birth. Point represents mean value with bar indicating 95% confidence interval.

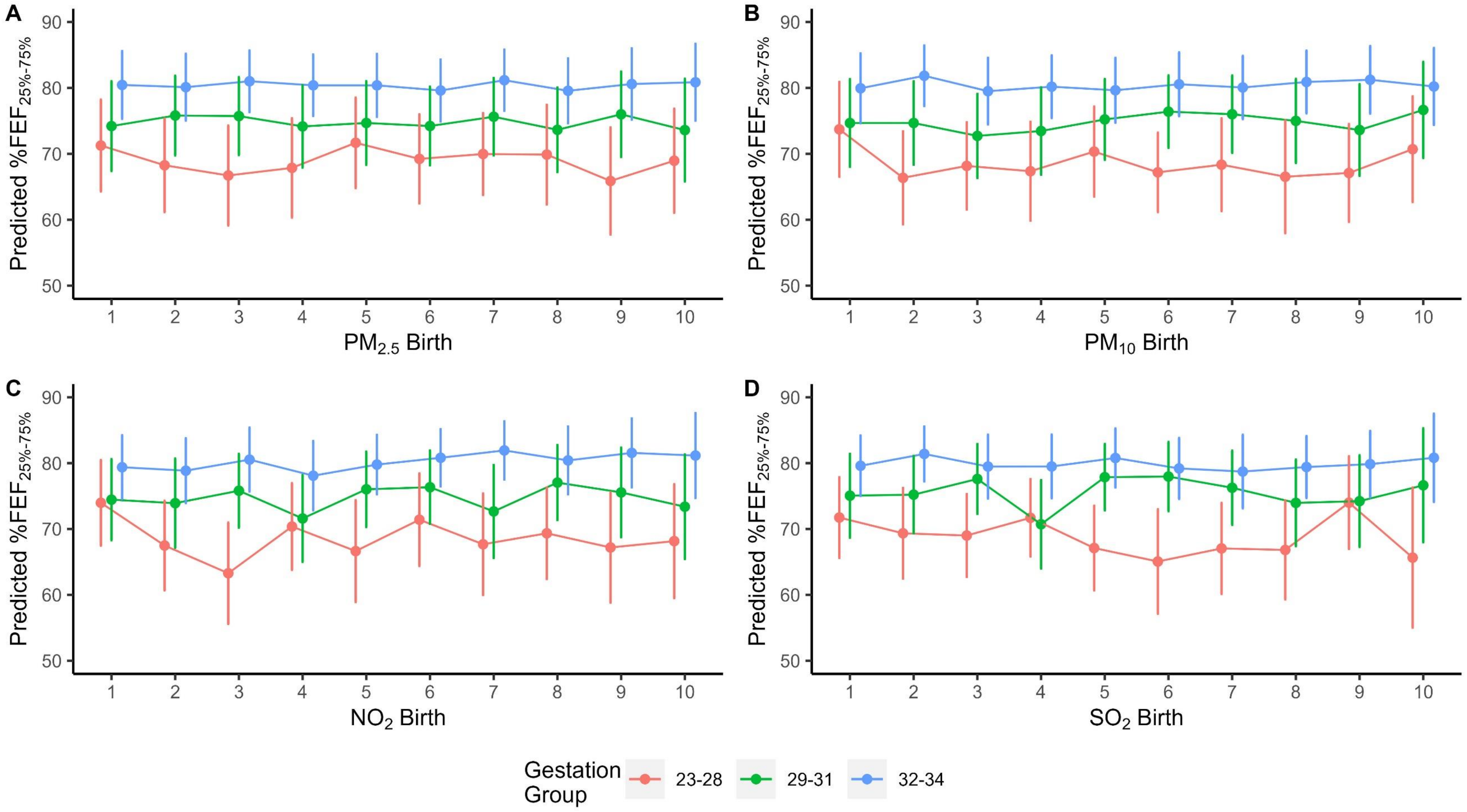

%FEF_{25-75}: Percentage predicted forced mid-expiratory flow; $PM_{2.5}$: Particulate matter with a diameter of 2.5μm; PM_{10}: Particulate matter with a diameter of 10μm; NO_2: Nitrogen dioxide; SO_2: Sulphur dioxide.

Supplementary Figure 4b: Predicted %$FEF_{25-75\%}$ over range of pollutant exposure by decile at time of spirometry. Point represents mean value with bar indicating 95% confidence interval.

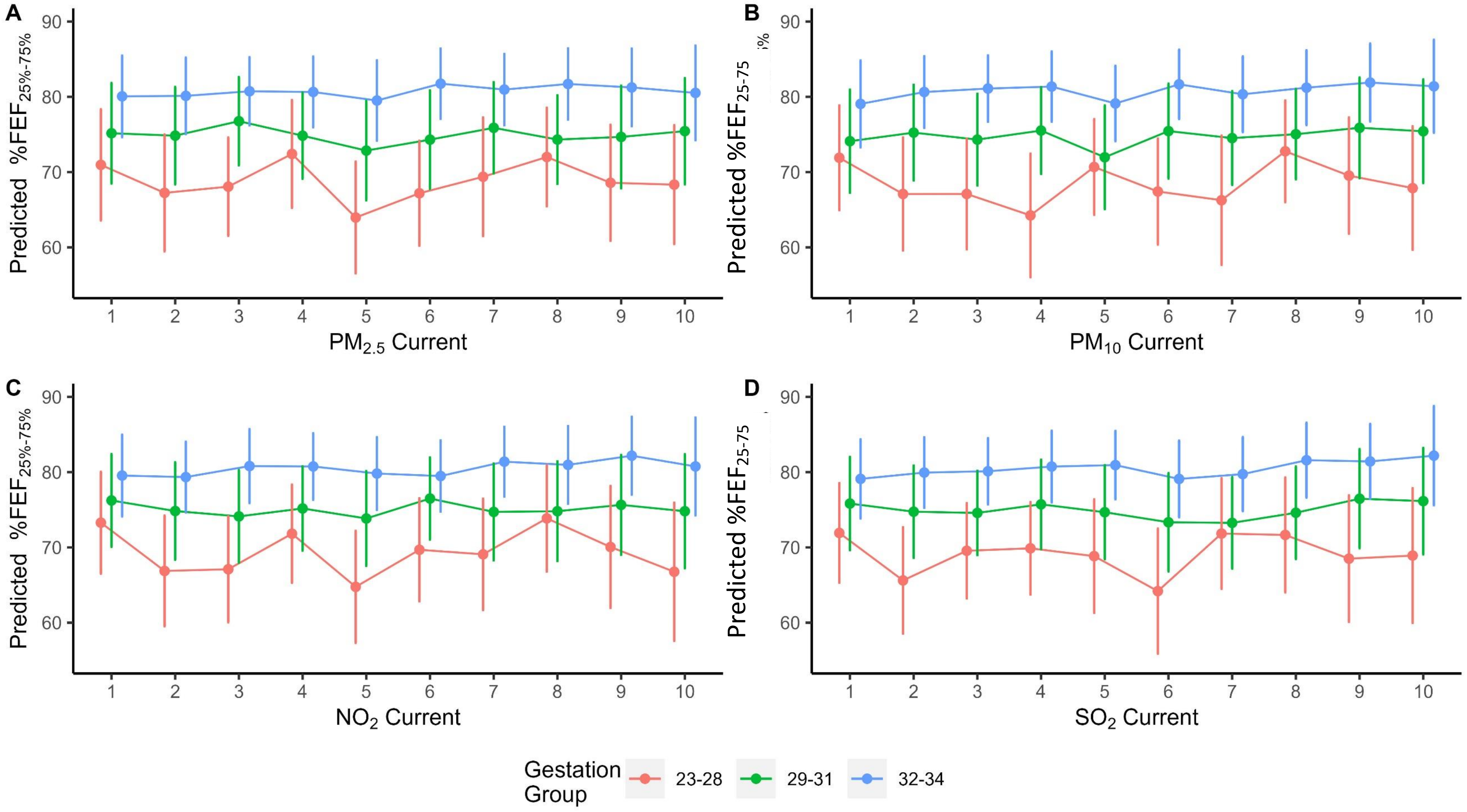

%FEF_{25-75}: Percentage predicted forced mid-expiratory flow; $PM_{2.5}$: Particulate matter with a diameter of 2.5µm; PM_{10}: Particulate matter with a diameter of 10µm; NO_2: Nitrogen dioxide; SO_2: Sulphur dioxide.

Supplementary Table 8: Pollutant levels by deciles for birth and time of spirometry.

Decile	$PM_{2.5}$ Birth				$PM_{2.5}$ Current			
	Mean	N	Minimum	Maximum	Mean	N	Minimum	Maximum
1	7.751	53	6.898	8.203	6.174	54	5.577	6.597
2	8.504	54	8.217	8.690	6.994	55	6.607	7.270
3	8.922	54	8.712	9.047	7.401	54	7.277	7.547
4	9.181	53	9.051	9.268	7.671	54	7.547	7.804
5	9.420	54	9.274	9.557	7.926	54	7.815	8.011
6	9.735	54	9.558	9.902	8.090	54	8.014	8.199
7	10.111	53	9.906	10.342	8.330	54	8.203	8.448
8	10.572	55	10.345	10.827	8.598	55	8.448	8.789
9	11.031	53	10.833	11.348	9.005	54	8.789	9.229
10	12.011	53	11.388	13.309	9.727	54	9.287	10.584
All	9.723	536	6.898	13.309	7.991	542	5.577	10.584
	PM_{10} Birth				PM_{10} Current			
	Mean	N	Minimum	Maximum	Mean	N	Minimum	Maximum
1	12.096	53	10.100	12.986	10.125	54	8.829	10.951
2	13.310	54	12.988	13.647	11.297	55	10.954	11.476
3	13.954	56	13.648	14.178	11.618	54	11.476	11.829
4	14.454	51	14.192	14.691	11.996	54	11.829	12.106
5	14.933	54	14.713	15.177	12.272	54	12.107	12.396
6	15.420	54	15.191	15.757	12.546	54	12.396	12.709
7	16.056	53	15.771	16.327	12.883	54	12.709	13.071
8	16.661	54	16.336	17.010	13.291	55	13.071	13.539
9	17.414	54	17.034	18.006	13.894	54	13.646	14.339
10	19.015	53	18.008	20.563	15.112	54	14.342	16.311
Total	15.329	536	10.100	20.563	12.503	542	8.829	16.311
	NO_2 Birth				NO_2 Current			
	Mean	N	Minimum	Maximum	Mean	N	Minimum	Maximum
1	6.576	53	4.479	8.396	4.995	54	3.803	6.735
2	9.683	54	8.455	10.600	8.230	55	6.783	9.253
3	11.464	54	10.635	12.148	9.920	54	9.344	10.557

Watkins WJ, *et al. Thorax* 2024; 79:553–563. doi: 10.1136/thorax-2023-220233

4	12.874	53	12.188	13.519	11.148	54	10.557	11.693
5	14.207	54	13.591	14.741	12.353	54	11.714	12.870
6	15.534	54	14.777	16.374	13.352	54	12.875	14.139
7	17.277	55	16.496	18.081	14.880	54	14.143	15.726
8	19.018	52	18.103	19.885	16.741	55	15.726	17.557
9	22.070	56	20.006	23.737	19.092	54	17.681	20.761
10	27.077	51	23.743	34.929	22.795	54	20.813	29.243
Total	15.550	536	4.479	34.929	13.347	542	3.803	29.243
	**SO_2 Birth**				**SO_2 Current**			
	Mean	N	Minimum	Maximum	Mean	N	Minimum	Maximum
1	1.175	53	0.809	1.461	0.944	54	0.549	1.220
2	1.665	54	1.463	1.823	1.414	55	1.230	1.571
3	1.980	54	1.829	2.134	1.723	54	1.584	1.805
4	2.259	53	2.147	2.426	1.861	54	1.805	1.896
5	2.575	54	2.435	2.693	1.979	54	1.903	2.046
6	2.827	54	2.695	2.959	2.087	54	2.046	2.126
7	3.111	53	2.964	3.271	2.214	54	2.127	2.313
8	3.416	55	3.273	3.642	2.408	55	2.316	2.510
9	4.060	53	3.651	4.701	2.610	54	2.513	2.723
10	6.791	53	4.747	15.893	3.423	54	2.723	8.819
Total	2.982	536	0.809	15.893	2.066	542	0.549	8.819

$PM_{2.5}$: Particulate matter with a diameter of 2.5µm; PM_{10}: Particulate matter with a diameter of 10µm; NO_2: Nitrogen dioxide; SO_2: Sulphur dioxide.

Watkins WJ, *et al. Thorax* 2024; 79:553–563. doi: 10.1136/thorax-2023-220233
